# Fluorescent Dynamic Covalent Polymers for DNA Complexation and Templated Assembly

**DOI:** 10.3390/molecules27196648

**Published:** 2022-10-06

**Authors:** Clément Kotras, Maxime Leclercq, Maxime Roger, Camille Bouillon, Antonio Recupido, Aurélien Lebrun, Yannick Bessin, Philippe Gerbier, Sébastien Richeter, Sébastien Ulrich, Sébastien Clément, Mathieu Surin

**Affiliations:** 1 Laboratory for Chemistry of Novel Materials, Center of Innovation and Research in Materials and Polymers (CIRMAP), University of Mons-UMONS, 7000 Mons, Belgium; 2 ICGM, UMR 5253, CNRS, Université de Montpellier, ENSCM, 34000 Montpellier, France; 3 IBMM, Université de Montpellier, CNRS, ENSCM, 34000 Montpellier, France; 4 Laboratoire de Mesures Physiques, 34000 Montpellier, France

**Keywords:** dynamic covalent polymers, fluorescence, DNA complexation, templated assembly

## Abstract

Dynamic covalent polymers (DCPs) offer opportunities as adaptive materials of particular interest for targeting, sensing and delivery of biological molecules. In this view, combining cationic units and fluorescent units along DCP chains is attractive for achieving optical probes for the recognition and delivery of nucleic acids. Here, we report on the design of acylhydrazone-based DCPs combining cationic arginine units with π-conjugated fluorescent moieties based on thiophene-ethynyl-fluorene cores. Two types of fluorescent building blocks bearing neutral or cationic side groups on the fluorene moiety are considered in order to assess the role of the number of cationic units on complexation with DNA. The (chir)optical properties of the building blocks, the DCPs, and their complexes with several types of DNA are explored, providing details on the formation of supramolecular complexes and on their stability in aqueous solutions. The DNA-templated formation of DCPs is demonstrated, which provides new perspectives on the assembly of fluorescent DCP based on the nucleic acid structure.

## 1. Introduction

Dynamic Covalent Chemistry (DCvC) encompasses a set of possible reactions enabling reversible covalent bond formation under thermodynamic control [1]. DCvC endows dynamic and adaptive properties which are now increasingly considered for designing self-healing, stimuli-responsive, and adaptive materials [2,3,4,5,6,7,8] such as Dynamic Covalent Polymers (DCPs) [9]. Alongside their interest in materials science, DCPs also bear a strong potential in biological applications such as sensing, imaging, delivery, and drug discovery [10,11,12,13,14,15,16,17]. Significantly, a recent report has demonstrated that a live cell-templated DCvC approach is effective in finding ligands binding the extracellular matrix, an important result in the search for new bioactive molecules targeting specific cells [18].

In particular, the complexation of nucleic acids by DCPs has attracted a large interest, in view of the strong potential for gene delivery applications arising from the switchable and/or adaptive dynamic self-assembly approach that enables the modulation of multivalent binding to nucleic acids [19,20,21]. A number of structurally distinct DCPs, such as dynamic amphiphiles [22,23,24,25], linear DCPs [26,27], dynamic covalent frameworks (DCFs) [13,19,20,28], and polymer/peptide scaffolded DCPs [29,30,31,32,33] have been shown to effectively complex different types of nucleic acids (DNA, siRNA [34]). It is only recently that evidence of the nucleic-acid templated adaptive self-assembly of DCPs have been reported [35,36] and exploited for the delivery of siRNA in cells [37,38]. Among DCPs, those bearing aromatic moieties represent a particular subclass for which hydrophobic interactions between aromatics can drive self-folding [39,40,41,42], that in return can influence the dynamic covalent polymerization process, or in which the intercalation in between the nucleic acid base pairs can impart specific binding properties [43]. Very recently, Reineke and coworkers reported copolymers incorporating quinine aromatic moieties as effective in both DNA complexation and delivery, and in imaging protein-induced unpackaging in cells [44].

To further progress towards DCPs for nucleic acid sensing and delivery, pH-sensitive and fluorescent units can be incorporated. Here, we report on the introduction of fluorescent moieties into acyl-hydrazone based DCPs. We describe the design and synthesis of the building blocks and characterize their self-assembly through DCvC. We study in detail the optical properties of both the building blocks and the new DCPs. Finally, we explore the complexation between DCPs and DNA, and the corresponding (chir)optical responses that pave the way towards the recognition and delivery of fluorescent DCPs as optical probes for nucleic acids, and more generally, toward medical imaging and biosensing. Finally, an example of DNA-templated formation of DCPs is demonstrated, which provides new perspectives to assemble fluorescent DCPs based on the nucleic acid structure.

## 2. Results and Discussion

### 2.1. Design and Synthesis of the Fluorescent Building Blocks

**Figure 1:**
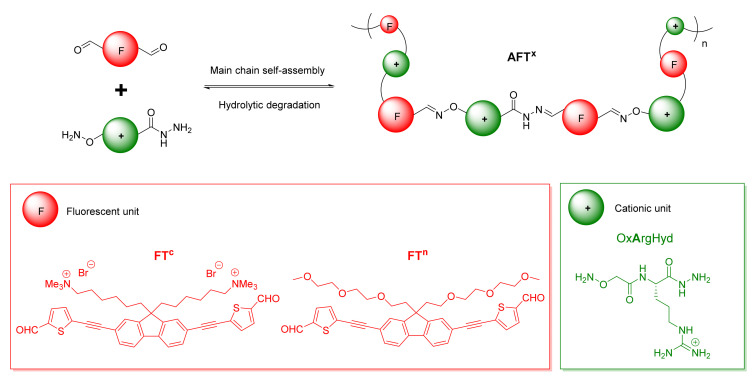
Schematic representation of the dynamic covalent polymer approach. The building blocks are fluorescent π-conjugated units (F, in red) and cationic arginine units (+, in green).

As fluorescent building blocks, our design strategy relies on the use of fluorene-based units as emissive π-conjugated moieties due to its high fluorescence quantum yield and good photostability (Figure 1) [45,46]. To redshift the absorption of these fluorene units in an optical region different from that where DNA is absorbed, the π-conjugation was extended by incorporating thiophenes and alkynes functionalities to the fluorene central core (Figure 1). The choice of the thiophene ring as π-electron bridge was motivated by its higher degree of aromaticity than other five-membered rings such as furan and its higher reactivity compared to benzene or furan rings which allows for further functionalization [47]. In addition, their incorporation in the molecular design of fluorene-based chromophores has been shown to enhance the optical properties and to increase both the maximum of absorption (λ_max_) and their two-photon absorption properties [48,49,50,51]. Finally, hexylammonium or ethylene glycol side chains were introduced at the 9-position of fluorene to evaluate the role of cationic or neutral side-groups in DCP formation and interaction with DNA (Figure 1).

The complementary partner is a modified amino acid bearing a hydrazide at the C terminus and an oxyamine at the N terminus that readily react with aldehydes at room temperature, either in DMSO [26,27] or in aqueous media [38] to form acylhydrazone and oxime linkages, respectively [26]. The building block **OxArgHyd** is derived from l-arginine, a cationic amino acid which is well-known for contributing to nucleic acids binding through electrostatic interactions and hydrogen bonds [52], forming a salt-bridge interaction with the phosphodiester groups of nucleic acids (Figure 1). **OxArgHyd** was synthesized as previously described [26,27].

**Scheme 1:**
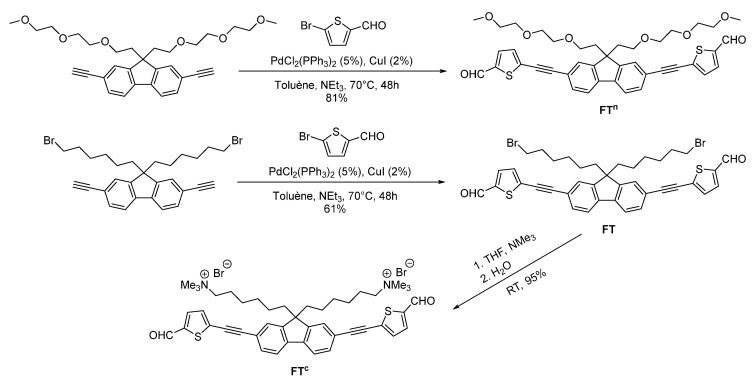
Synthesis of fluorene-based monomer building blocks.

The synthesis of the fluorescent π-conjugated monomers was achieved following the synthetic pathway described in Scheme 1. **FT** and **FT^n^** were prepared through a Sonogashira cross-coupling reaction between 2,7-diethynyl-9H-fluorene bearing either 3-bromohexyl or ethylene glycol side chains, respectively, and 5-bromo-2-thiophenecarboxaldehyde. **FT** and **FT^n^** were, respectively, isolated in 61% and 81% yield after purification by column chromatography on silica gel.

The presence of the carboxaldehyde end group was confirmed by IR spectroscopy with the presence of the carbonyl (C=O) stretching vibration band around 1650 cm^−1^ and the C-H stretch around 2850 cm^−1^ (Figure S9). A deshielded proton signal around 9.9 ppm was also observed in the ^1^H NMR spectra of **FT** and **FT^n^** in CDCl_3_, confirming unambigously the presence of the aldehyde protons. Finally, high-resolution ASAP mass spectrometry (positive mode) of **FT^n^** showed the presence of the molecular ion [M]^●+^ at *m*/*z* = 726.2314 Da in agreement with the calculated one (calcd *m*/*z* = 726.2321 Da), see Figure S12. **FT** was then converted quantitatively to the corresponding ammonium species **FT^c^** by reaction with trimethylamine in THF at room temperature. The presence of the trimethylammonium groups was confirmed by the appearance of a singlet at 2.97 ppm in the ^1^H NMR spectrum and at 65.1 ppm in the ^13^C{^1^H} NMR spectrum, corresponding to the two NMe_3_^+^ groups. Satisfyingly, MALDI-TOF mass spectrometry (positive mode) features the molecular peak at m/z = 797.2805 Da, which is in good agreement with the calculated one (calcd *m*/*z* = 797.2810 Da) (Figure S8).

### 2.2. Synthesis of Fluorescent DCPs

The self-assembly by polycondensation of **OxArgHyd** and **FT^n^** building blocks was first carried out at room temperature, as previously described [22,23,24,25], in DMSO in a wide range of concentrations. DMSO was selected as it was found to be a good solvent at all the tested concentrations. The self-assembly under thermodynamic control is highly sensitive to concentration yielding macrocycles at low concentration and DCPs above a threshold concentration [22,23,24,25]. DOSY-NMR analyses of reactions carried out at 1, 10, and 50 mM of each building block in stoichiometric amount indicate a typical increase in hydrodynamic diameter compared to the monomer precursor **FT^n^** (R_h_ = 8 Å) (Table 1). This can be interpreted by a change in constitution, going from oligomers at low concentration to DCPs at higher concentration; a typical behavior for step-growth polycondensations. For comparison, a DOSY NMR analysis of **AFT^c^** at 10 mM was also performed revealing that, as expected due to the rather similar structure, species of similar size as **AFT^n^** and larger than monomers are formed (R_h_ = 24 Å vs. R_h_ = 9 Å for **FT^c^**, see Table 1).

For these reasons, we decided to further investigate the effect of the concentration on only **AFT^n^**. As such, a sample of **AFT^n^** was also prepared at 100 mM but unfortunately, could not be analyzed by DOSY-NMR due to a significant increase in viscosity that further confirms the formation of high molecular weight species. Nevertheless, this sample could be analyzed by MALDI-TOF mass spectrometry and ^1^H NMR spectroscopy. The MS analysis of **AFT^n^**, prepared in DMSO at 100 mM, clearly shows the formation of oligomeric species (Figure S15). The formation of oligomeric species was also confirmed by the analysis of the ^1^H NMR spectrum of this sample in DMSO-*d*_6_. ^1^H NMR spectrum of **AFT^n^** reveals the presence of the NH protons of the acylhydrazone groups between 11.5 and 12 ppm as well as that of remaining aldehydic protons at 9.91 ppm. By the relative integration of this aldehyde end-group and methylene peaks at 2.66 ppm, a degree of polymerization of around 10 was estimated, (Figure S16) confirming the formation of oligomers [53].

### 2.3. Study of (Chir)optical Properties of DCPs

**Figure 2:**
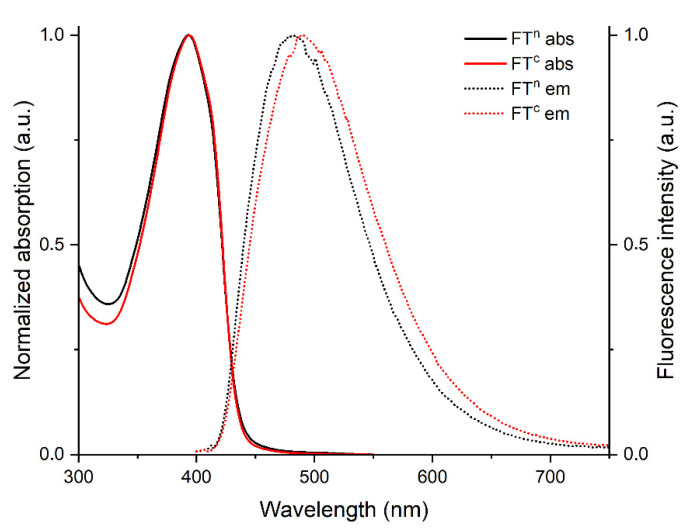
Normalized UV-Vis absorption spectra (plain curves) and emission spectra (dashed curves) of **FT^c^** and **FT^n^** in DMSO.

The (chir)optical properties of the building blocks and DCPs were investigated by UV-Vis absorption, circular dichroism (CD) and fluorescence spectroscopies, both in DMSO and in aqueous solutions (Tris-EDTA buffer at pH 7.4). Figure 2 and Table 2 show the results for solutions of **FT^n^**/**FT^c^** in DMSO. Both chromophores show an intense absorption ranging in the region 330 nm–440 nm, with maxima around 395 nm. The characteristic maximum absorption wavelengths and molar extinction coefficients for the two compounds are listed in Table 2. In DMSO, **FT^n^** and **FT^c^** exhibited rather similar emission profile with maxima of 481 nm and 487 nm, respectively. The fluorescence quantum yields, determined using an integrating sphere, are relatively high, around 0.5 for both compounds.

**Figure 3:**
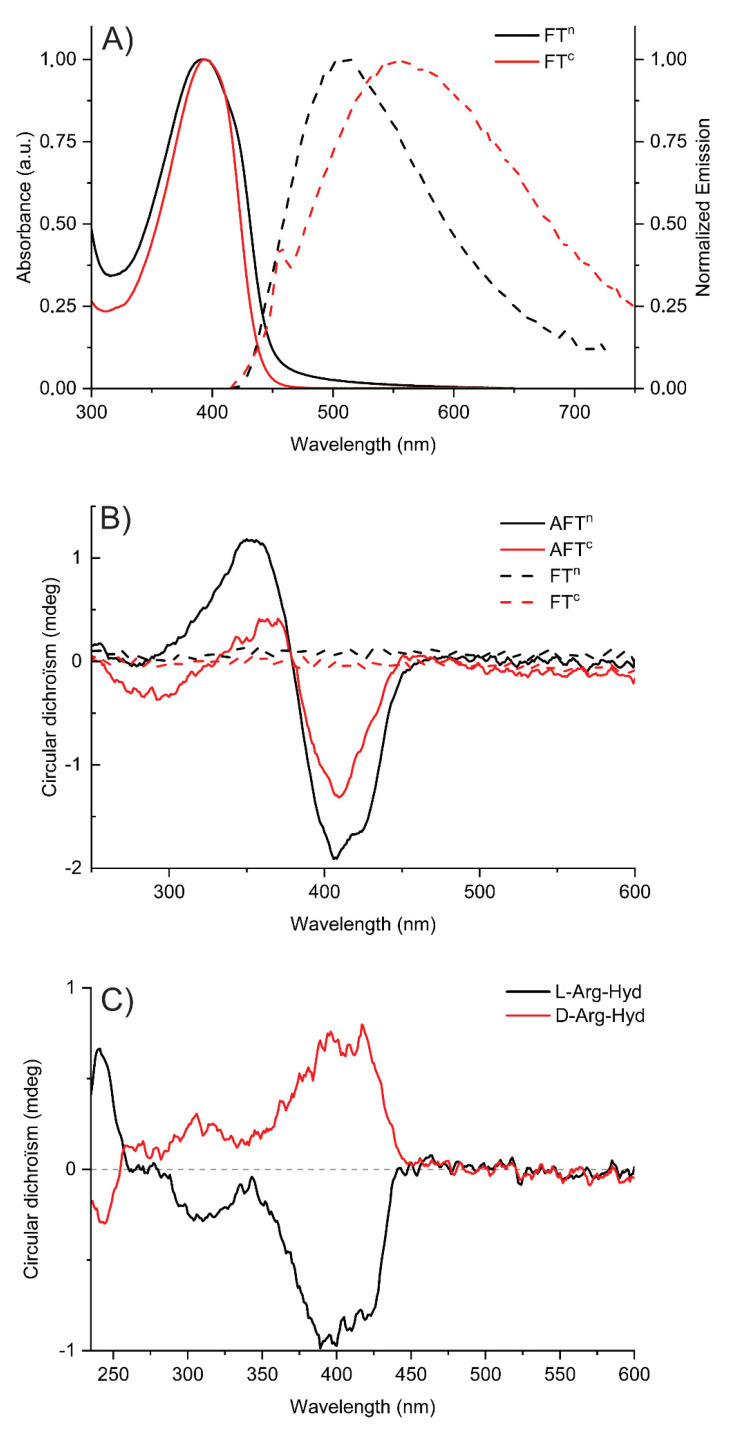
Spectroscopic analysis for 10 μM aqueous solutions in TE buffer (pH 7.4). (**A**) Normalized UV-Vis absorption spectra (plain curves) and emission spectra (dashed curves) of **FT^c^** and **FT^n^** in TE buffer. (**B**) CD spectra of **FT^c^**, **FT^n^**, **AFT^c^** and **AFT^n^**. (**C**) CD spectra of ***l*-Arg-Hyd** and ***d*-Arg-Hyd** conjugates of **FT^n^**.

In aqueous solutions (TE buffer, used for subsequent experiments with DNA), the UV-Vis absorption spectra (Figure 3A) are rather similar than those in DMSO, peaking around 395 nm. However, the fluorescence spectra exhibit large red-shifts of **λ_em_** compared to solutions in DMSO with maximum at 504 nm and 549 nm for **FT^n^** and **FT^c^**, respectively. This red-shift is more pronounced for **FT^c^**, pinpointing the differences of conformation/assembly for neutral or charged DCPs in aqueous solutions. The chiroptical spectra of the DCPs **AFT^c^** and **AFT^n^** in aqueous solutions (TE buffer) are markedly different from those of fluorescent building blocks **FT** (Figure 3B). While the achiral **FT^c^** and **FT^n^** both show indeed a zero CD signal, DCPs show a bisignate CD signal in the region 330 nm–440 nm. To evaluate the role of the amino acid chirality, model compounds were synthesized by combining **FT^n^** fluorescent units with either ***l*-ArgHyd** or ***d*-ArgHyd** (see synthesis in Figure S17) and studied by CD spectroscopy. CD spectra showed induced CD signals in the fluorene absorption region around 375–425 nm (Figure 3C). This induced CD signal is positive with ***d*-ArgHyd** and negative with ***l*-ArgHyd**, thus demonstrating the chirality transfer from the modified amino acids toward the achiral central fluorophore.

Interestingly, the (chir)optical properties of **AFT^c^** and **AFT^n^** showed some dependence on the presence of salts that is specific to the DCPs and not seen on the fluorophore alone. Indeed, by adding an increasing concentration of guanidinium chloride, a chaotropic salt that weakens hydrophobic interactions, a gradual decrease in the intensity of the CD spectra is observed (Figure S19B). Conversely, an increasing concentration of ammonium sulfate, a kosmotropic salt that increases hydrophobic interactions, leads to a decrease in the fluorescence emission (Figure S20C). Altogether, those results indicate that hydrophobic interactions and/or π-stacking interactions of the fluorophores are at play in the formation of these folded DCPs. The stability of DCPs was further assessed by variable-temperature CD spectroscopy (from 20 °C to 86 °C, see Figure S21). For **AFT^n^**, CD signals weaken at 34 °C and vanish around 45 °C (Figure S20B). At the end of the temperature ramping, a precipitate is observed and cooling the solution does not allow for a return to a dissolved state. In contrast, for **AFT^c^**, the CD signals were barely modified during the temperature ramping, indicating that the structuring of the polymer in the solution seems to be preserved even at high temperatures (85 °C, see Figure S21A), and no precipitate was observed in this case.

### 2.4. Supramolecular Complexes of DCPs and DNA

**Figure 4:**
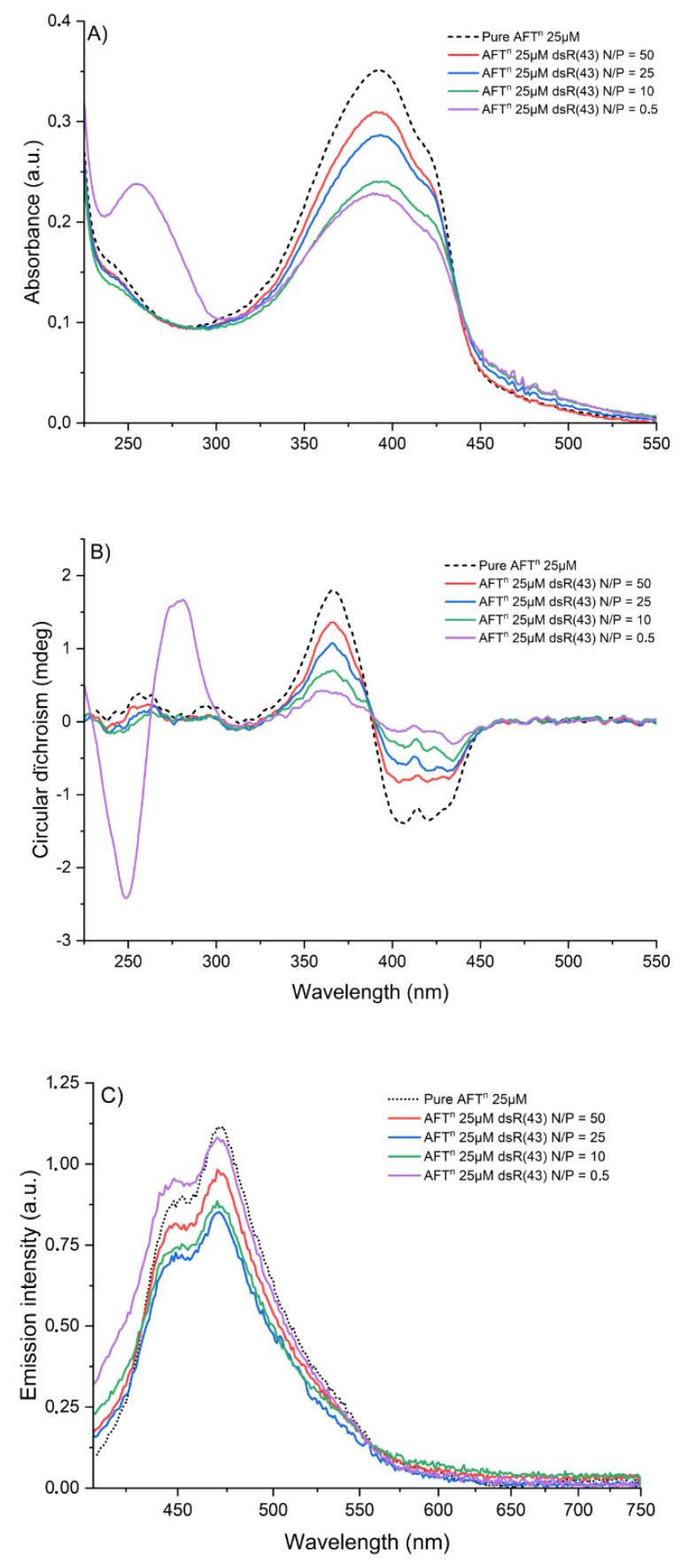
UV-Vis absorption (**A**), CD (**B**) and emission (**C**) spectra of 25 µM **AFT^n^** solutions in TE buffer (pH 7.4) upon addition of dsR(43) DNA.

The complexation between DNA and DCPs was studied by (chir)optical spectroscopies and gel electrophoresis. Each DCP solution was titrated by successive additions of DNA and monitored by UV-Visible absorption, CD and fluorescence spectroscopies, keeping the DCP concentration constant (at 10 µM). These titrations were carried out for 3 types of double-stranded DNA sequences: two short sequences dsR20 and dsR43, respectively 20 and 43 base pairs (see sequence in Table S1), and calf thymus DNA, which comprises approximately 12 million base pairs. The short sequences were selected for the sake of comparison with our previous studies on the complexation of identical DNA sequences with π-conjugated polymers (see below) [54,55]. Figure 4 shows the evolution of spectroscopic signals during the titration, shown by following the charge (+)/(−), also referred to as N/P ratio as reported in DNA-binding experiments. For both solutions of **AFT^c^** and **AFT^n^**, the addition of DNA, whatever its sequence or length, leads to an increase in the intensity of the UV-Vis absorption bands between 225 nm and 325 nm related to the increasing the concentration of DNA in the solution (Figure 4A for **AFT^n^** and Figures S22–S23). In the visible part of the spectra, a significant decrease in the absorption bands is observed upon addition of DNA. CD spectra (Figure 4B for **AFT^n^** and Figure S22–S23) show a decrease in intensity of the signals between 325 nm and 450 nm, which may indicate a decrease in exciton coupling between the chromophores upon addition of DNA. The decrease in the UV-Vis absorption and CD spectra related to DCPs is even more pronounced when N/P ratio varies (Figure 4A,B and Figure S22–S23). This drop in the signal intensities could be explained by the complexation of DNA by the DCP, which could modify the chiral organization of DCP compared to the pure DCP in aqueous solution. We also noticed the appearance of aggregates when a certain N/P molar ratio was exceeded, depending on the compound and DNA type.

The same titration experiments were monitored by fluorescence spectroscopy, as shown in Figure 4C for **AFT^n^**. After addition of a low amount of dsR43 DNA (at high N/P ratios, e.g., N/P = 25), we observed a ~25% decrease in the intensity of the emission peaks at 448 and 470 nm compared to pure **AFT^n^**. However, at N/P of 5, the intensity of signals slightly increases, up to a N/P of 0.5, reaching the intensities observed for pure **AFT^n^**. This behavior suggests that during the addition of DNA, the stacking of π-conjugated moieties is modified. During the first additions of DNA (at N/P ≥ 25), the extinction of the fluorescence could be induced by the stacking of the π-conjugated units, causing the extinction of the fluorescence by aggregation. Then, when the quantity of DNA increases, the increase in the emission intensity could be related to an unfolding of the polymer chains during their interaction with more DNA strands or a dissociation of the non-emissive aggregates via the interaction between DCP and DNA. This hypothesis could also be reinforced by the emission spectra recorded during the successive additions of calf thymus DNA (Figure S21). Indeed, double-stranded oligonucleotides such as dsR(20) and dsR(43) DNAs being shorter, they are rather linear rods in solution, whereas the much longer calf thymus DNA (12 million base pairs) tends to fold up in the form of a coil in solution, possibly leading to a higher extent of aggregation-induced fluorescence extinction through interactions between DCPs chains.

**Figure 5:**
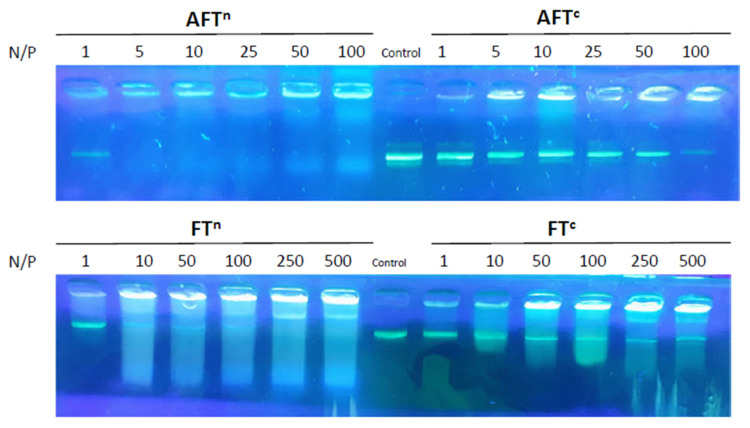
Electrophoresis analysis of pDNA complexation at N/P = 1–500 by the DCPs **AFT^n^** and **AFT^c^** (**top**) compared to the fluorophores **FT^n^** and **FT^c^** (**bottom**). Control refers to pDNA alone.

Gel electrophoresis was used to probe the interaction with plasmid DNA (pDNA) through an electrophoretic mobility shift assay (Figure 5). Both DCPs show effective complexation starting at N/P = 5. While a complete complexation is observed with neutral **AFT^n^**, a more gradual complexation is seen with **AFT^c^**. On the other hand, both fluorophores show very different profiles in presence of pDNA. Although the complexation is also observed, the constant formation of streaks in all conditions point to a very different mechanism of interaction, which could indicate interactions within base pairs, or could also be the result of the poor solubility of those building blocks alone in aqueous media.

### 2.5. DNA-Templated Formation of DCPs

**Figure 6:**
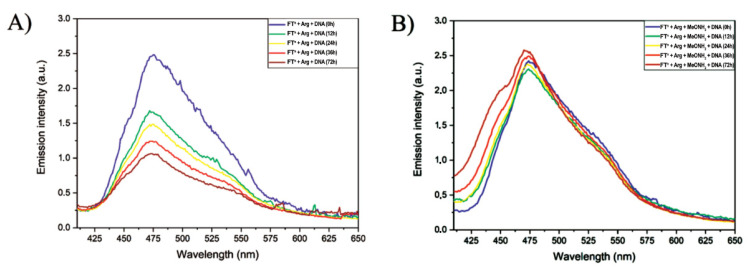
(**A**) Emission spectra (λ_exc_ = 385 nm) of a solution of **FT^n^** and **OxArgHyd** (both at 10 µM) in presence of calf thymus DNA with a charge ratio N/P = 5, from 0 to 72 h; (**B**) Emission spectra (λ_exc_ = 385 nm) of the same solution in presence of 100 eq. of methoxyamine. Solutions are in an aqueous TE buffer (pH 7.4).

The use of chemical ligation reactions, such as acylhydrazone and oxime formation [56,57], can be carried out in aqueous media in mild conditions compatible with the presence of nucleic acids and opens the door toward the in situ formation of DCPs. The nucleic acids can act as templates and guide the formation of self-fitted DCPs at an unusually low concentration in which DCPs would otherwise not form [34,35,36,37,38]. Here, we tested the in situ self-assembly of both building blocks in the presence of CT-DNA and monitored the outcome by CD and fluorescence spectroscopies. For this, the **FT^n^** monomer was introduced into a cuvette at a concentration of 10 μM in a TE buffer solution at pH 7.4; then, double-stranded DNA extracted from calf thymus was added directly to the cuvette in order to place itself at N/P = 5. Then, the second monomer **OxArgHyd** was added to the mixture at the same concentration as the fluorescent monomer and the process was monitored by recording emission spectra after 12 h, 24 h, 36 h, 48 h and 72 h after the addition of **OxArgHyd**. The results show a decrease in the fluorescence emission with time, similar to what is found during the complexation of DNA by the pre-formed DCPs (Figure 6A vs. Figure 4C). A negative control experiment was also conducted by adding 100 equivalents of methoxyamine before the addition of **OxArgHyd** to allow for the formation of oxime **Ox-FT^n^-Ox** (Scheme S1). The addition of methoxyamine in large excess leads to a displacement of the thermodynamic equilibrium and prevents the formation of DCPs by consuming the fluorene bisaldehyde brick present in the solution (Figure 6B). This indicates that the decrease in the fluorescence in the experiments without methoxyamine points toward the templated self-assembly of those DCPs.

The monitoring of this process by CD spectroscopy confirms the templating role of CT-DNA on the formation of DCPs (Figure S24). The CD signals obtained after 36 h are markedly different from those obtained when mixing DCPs and DNA. When templated by DNA, the formation of DCP exhibits positive peaks in the spectral range where the π-conjugated moieties absorb. This is in contrast to the bisignate CD signals observed for the mixture DNA/DCP (see Figure 4B), which indicates that DNA-templated assembly lead to a different chiral organization of DCP compared to complexation of a previously formed DCP mixed to DNA (Figure S23 vs. Figure 4B).

## 3. Materials and Methods

### 3.1. Materials

Reactions were performed under argon using oven-dried glassware and Schlenk techniques. 9,9-bis(6′-bromohexyl)-2,7-diethynyl-9H-fluorene, 2,7-diethynyl-9,9-bis[2-[2-(2-methoxyethoxy)ethoxy]ethyl]-9H-fluorene were prepared according to literature procedures [58,59]. Bis(triphenylphosphine)palladium(II) dichloride (PdCl_2_(PPh_3_)_2_, 98%), copper iodide (CuI, 98%), triethylamine (99%) were purchased from Fluorochem (Hadfield, UK). 5-bromo-2-thiophenecarboxaldehyde (97%) and trimethylamine (2.0 M in THF) were purchased from Alfa Aesar (Haverhill, MA, USA). Solvents were obtained from commercial suppliers and used as received. Dry toluene and THF were obtained from a solvent purification system, PureSolve MD5 purchased from Inert Technology (Amesbury, MA, USA).

### 3.2. Characterization Methods

The FTIR analysis was performed on a Perkin Elmer (Waltham, MA, USA) Spectrum Two™ FT-IR Spectrometer from powders in the attenuated total reflectance (ATR) mode. NMR spectra were recorded at the Laboratoire de Mesures Physiques (LMP) of the University of Montpellier (UM) using either a Bruker Avance III 500 MHz or 600 MHz spectrometers (Bruker, Fallenden, Switzerland) and were calibrated to TMS on the basis of the relative chemical shift of the solvent used as an internal standard. Moreover, 2D DOSY NMR spectra were recorded on a Bruker Avance III 600 (Bruker, Fallenden, Switzerland) spectrometer equipped with a 5-mm TCI Prodigy Z-gradient cryoprobe. The standard gradient amplifier (GREAT 1/10-E (Bruker, Fallenden, Switzerland)) generates a maximum gradient strength of 65.7 Gauss cm^−1^ at a current of 10 A. The gradient strengths were calibrated using a sample of D_2_O 99.96% deuterated from Eurisotop (Saint-Aubin, France). All diffusion measurements were performed with double stimulated echo and bipolar gradient pulses for convection compensation and Eddy current delay (dstebpgp) in a pseudo 2D mode and processed with T1/T2 module from Topspin 3.6.2 (Bruker, Fallenden, Switzerland). For each experiment, 8 dummy scans and 32 scans were used with a relaxation delay of 3 s. Diffusion Time Δ was fixed between 0.1 s and 0.3 s and the gradient strength δ ranged between 2.8 and 5.0 ms depending on the molecule size. Sinusoidal shapes were used for the gradients and a linear gradient ramp with 16 increments between 2 and 95% was applied for the diffusion relevant gradients. The diffusion coefficients were calculated with T1/T2 module from Topspin 3.6.2 (Bruker, Fallenden, Switzerland). All diffusion coefficients were within an error range of ±5%. Mass accuracy measurement for **FT** and **FT^c^** were performed by using a Waters QTOF (Waters, Antwerpen, Belgium) Premier mass spectrometer equipped with Matrix-assisted laser desorption/ionization source. A Nd-YAG laser of 355 nm with a maximum pulse energy of 65 μJ delivered to the sample at 50 Hz repeating rate is used. Time-of-flight mass analyses is performed in the reflection mode at a resolution of about 10 k (*m*/*z* 569). Trans-2-(3-(4-tert-butyl-phenyl)-2-methyl-2-propenylidene)malononitrile (DCTB) is used as the matrix and is prepared as a 40 mg/mL solution in chloroform. The matrix solution (1 μL) is applied to a stainless-steel target and air-dried. Samples are dissolved in chloroform to obtain 1 mg/mL solutions. Furthermore, 1 μL aliquots of these solutions are applied onto the target area (already bearing the matrix crystals) and air-dried. Exact mass is determined by using poly(ethylene glycol) as an internal reference. The mass spectra of **FT^n^** were recorded on a Synapt G2-S (Waters, Saint-Quentin-en-Yvelines, France) spectrometer based on a quadrupole Time of Flight (Q-TOF) analyser and equipped with an Atmospheric Solids Analysis Probe (ASAP) in positive mode between 100 and 1500 Da. The mass spectra of **AFT^n^** were recorded on a Rapiflex (Bruker, Billerica, MA, USA) with Matrix-Assisted Laser Desorption Ionization (MALDI) source and TOF analyzer. The mass spectrum was recorded in positive mode between 500 and 15,000 Da in linear mode using α-cyano-4-hydroxycinnamic acid (HCCA) as matrix. DMSO was not removed prior to mixing with the MALDI-TOF matrix.

### 3.3. Synthesis of Fluorescent Building Blocks FT^n^ and FT^c^

**FT**. 2,9-bis(6′-bromohexyl)-2,7-diethynyl-9H-fluorene (538 mg, 1 mmol), PdCl_2_(PPh_3_)_2_ (35 mg, 0.05 mmol), CuI (10 mg, 0.02 mmol) were placed in a 100 mL two-necked round-bottom flask with toluene (25 mL). Additionally, 5-bromo-2-thiophenecarboxaldehyde (478 mg, 297 μL, 2.5 mmol) in triethylamine (5 mL) was added dropwise. The reaction mixture was heated at 70 °C during 8 h. The solution was evaporated and the residue was purified by column chromatography on silica gel using cyclohexane/CH_2_Cl_2_ (20:80 to 100) as eluent. Yield: 61%. FTIR-ATR: υ = 2837, 2787 (C_sp2_-H, aldehyde), 2195 (C≡C), 1662 (C=O) cm^−1^. ^1^H NMR (500.17 MHz, CDCl_3_, 298K): δ = 9.91 (s, 2H, CHO), 7.79–7.70 (m, 4H), 7.59 (d, 2H, ^3^*J*_H-H_ = 7.0 Hz), 7.54 (s, 2H), 7.38 (d, ^4^*J*_H-H_ = 4.0 Hz, 2H), 3.31 (t, 4H, ^3^*J*_H-H_ = 6.2 Hz, CH_2_Br), 2.06–1.98 (m, 4H), 1.67 (quint, 4H, ^3^*J*_H-H_ = 6.8 Hz), 1.28–1.06 (m, 8H), 0.68–0.58 (m, 4H) ppm. ^13^C{^1^H} NMR (125.77 MHz, CDCl_3_, 298K): δ = 182.5 (C=O), 151.2, 144.1, 141.5, 136.3, 133.1, 132.6, 131.3, 126.1, 121.2, 120.6, 98.9, 82.9, 55.5, 40.3, 34.1, 32.8, 29.2, 27.9, 23.7 ppm. HR-MS (MALDI-TOF^+^): *m*/*z* calcd for C_39_H_36_Br_2_O_2_S_2_ [M]^•+^ 758.0523, found 758.0525 Da.

**FT^c^**. FT (100 mg, 0.13 mmol) was placed into a 100 mL two-necked round-bottom flask and then, dissolved in THF (5 mL). A volume of 7 mL of a solution of trimethylamine (2.0 M in THF) were added and the mixture was stirred at room temperature for 24 h. Water (3 mL) was added to dissolve the precipitate and 3 mL of the solution of trimethylamine were added. The reaction mixture was stirred for 8h. The solvent was then evaporated and the residue was dissolved in the minimum amount of methanol. **FT^c^** was then precipitated in diethyl ether (250 mL), filtered and dried under vaccum. Yield: 95% (109 mg). FTIR-ATR: υ = 2839, 2786 (C_sp2_-H, aldehyde), 2194 (C≡C), 1650 (C=O) cm^−1^. ^1^H NMR (500.17 MHz, DMSO-*d*_6_, 298K): δ = 9.95 (s, 2H), 8.08 (d, ^4^*J*_H-H_ = 4.0 Hz, 2H), 8.00 (d, ^3^*J*_H-H_ = 7.9 Hz, 2H), 7.80 (s, 2H), 7.64 (dd, ^5^*J*_H-H_ = 1.4 Hz, ^3^*J*_H-H_ = 7.9 Hz, 2H), 7.38 (d, ^3^*J*_H-H_ = 4.0 Hz, 2H), 3.19–3.11 (m, 4H), 2.97 (s, 18H), 2.15–2.04 (m, 4H), 1.46 (quint, ^3^*J*_H-H_ = 7.7 Hz, 4H), 1.10 (quint, ^3^*J*_H-H_ = 7.0 Hz, 4H), 1.02 (quint, ^3^*J*_H-H_ = 7.0 Hz, 4H), 0.48 (quint, ^3^*J*_H-H_ = 7.6 Hz, 4H). ^13^C{^1^H} NMR (125.77 MHz, DMSO-*d*_6_, 298K): δ = 184.1 (C=O), 151.0, 143.9, 141.1, 138.2, 134.0, 131.1, 130.4, 126.0, 121.2, 120.1, 98.2, 82.7, 65.1, 55.2, 52.0, 28.5, 25.3, 23.1, 21.8 ppm. HR-MS (MALDI-TOF^+^): m/z calcd for C_45_H_54_N_2_O_2_S_2_Br [M]^+^ 797.2810, found 797.2805 Da.

**FT^n^**. 2,7-Diethynyl-9,9-bis[2-[2-(2-methoxyethoxy)ethoxy]ethyl]-9H-fluorene (506 mg, 1 mmol), PdCl_2_(PPh_3_)_2_ (35 mg, 0.05 mmol), CuI (10 mg, 0.02 mmol) were placed in a 100 mL two-necked round-bottom flask with toluene (25 mL). 5-bromo-2-thiophenecarboxaldehyde (478 mg, 297 μL, 2.5 mmol) in triethylamine (5 mL) was added dropwise. The reaction mixture was heated at 70 °C during 8 h. The solution was evaporated and the residue was purified by column chromatography on silica gel using CH_2_Cl_2_/MeOH (from 100:0 to 95:5 *v*/*v*) as eluent. Yield: 81% (orange powder). FTIR-ATR: υ = 2838, 2788 (C_sp2_-H, aldehyde), 2198 (C≡C), 1656 (C=O) cm^−1^. ^1^H NMR (500.17 MHz, CDCl_3_, 298K): δ = 9.89 (s, 2H, CHO), 7.70 (d, 2H, ^3^*J*_H-H_ = 3.9 Hz, C-H_thi__ényl_), 7.70 (d, 2H, ^3^*J*_H-H_ = 7.9 Hz), 7.62 (d, 2H, ^4^*J*_H-H_ = 1.4 Hz), 7.56 (dd, 2H, ^3^*J*_H-H_ = 7.9 Hz, ^4^*J*_H-H_ = 1.4 Hz), 7.36 (d, ^3^*J*_H-H_ = 3.9 Hz, 2H); 3.53–3.45 (m, 8H), 3.40–3.36 (m, 4H), 3.32 (s, 6H, OCH_3_), 3.23–3.18 (m, 4H), 2.82–2.76 (m, 4H); 2.42 (t, 4H, ^3^*J*_H-H_ = 7.4 Hz) ppm. ^13^C{^1^H} NMR (125.77 MHz, CDCl_3_, 298K): δ = 182.5 (C=O), 149.9, 144.1, 140.8, 136.3, 133.0, 132.7, 131.5, 126.7, 121.4, 120.6, 98.6, 83.1, 72.0, 70.6, 70.2, 67.0, 59.2, 51.8, 39.7, 29.8 ppm. HRMS (ASAP^+^ TOF) m/z calcd for C_41_H_42_O_8_S_2_ [M]^•+^ 726.2321, found 726.2314.

### 3.4. Synthesis of the DCPs AFT^n^ and AFT^c^

Stock solutions of fluorescent monomers (**FT^n^**, **FT^c^**) and **OxArgHyd** at 100 mM were prepared in DMSO or DMSO-d_6_. An aliquot of these stock solutions of fluorescent and **OxArgHyd** monomers was taken to achieve the desired concentration (1 mM, 10 mM and 50 mM) of the reaction mixture in DMSO or DMSO-d_6_ (3 mL) and the reaction was stirred at room temperature for 5 days. Control experiments with MeONH_2_ and **L-Arg-Hyd** were carried out similarly.

### 3.5. Study of the Optical Properties

UV-Visible absorption spectra were performed at 298K on a JASCO V-750 spectrophotometer in 10 mm quartz cells (Hellma-France, Paris, France). All of the extinction coefficients were determined by preparing solutions of **FT^n^** and **FT^c^** monomer building blocks at different concentrations exploiting the Beer-Lambert relationship in the linear range (A~0.2–0.8). Emission spectra were recorded at 25 °C on a fluorescence spectrometer (FS920) (Edinburgh Instruments, Livingston, UK) equipped with a calibrated photomultiplier in a Peltier (air cooled) housing (R928P) (Hamamatsu Photonics France, Massy, France), with a 450 W continuous xenon arc lamp as the excitation source for steady-state photoluminescence measurements using a quartz cuvette with 1.0 cm excitation path length. Emission and excitation spectra were corrected for the wavelength response of the system using correction factors supplied by the manufacturer. The quantum yields of **FT^n^** and **FT^c^** in solution were determined using an integrating sphere (120 mm diameter) of Edinburgh Instruments under air.

### 3.6. Study of the Chiroptical Properties

UV-Vis, CD, and emission spectra of DCP/DNA complexes were recorded using a ChirascanTM Plus CD Spectrometer from Applied Photophysics. The measurements were performed using 2 mm Suprasil quartz cells. The spectra were recorded between 230 and 650 nm, with a bandwidth of 1 nm, time per point 0.5 s and two repetitions. The buffered water solvent reference spectra were used as baselines and were automatically subtracted from the CD spectra of the samples. Stock solutions of monomers or polymers (10 mΜ) in DMSO were prepared. A volume of 3 μL of these stock solutions was added to the buffer water solvent to obtain a 10 μM solution (remaining percentage of DMSO in the final solution (0.1% *v*/*v*)). The buffer water solvent was Tris-EDTA which was prepared from 1 M Tris-Cl and 0.5 M EDTA to achieve a 10 mM Tris-Cl and 1 mM EDTA final buffer at pH 7. The selected DNA was successively added to the monomer or polymer solution in TE buffer to achieve the defined N/P.

### 3.7. Electrophoretic Mobility Shift Assay (EMSA) with pDNA

A total of 100 ng of 5.7 kilobase pair expression vector, pET-15b (Novagen) were mixed with appropriate amounts of polymer to achieve the desired N/P ratio, and diluted in TE buffer (20 mM Tris-acetate/0.5 mM EDTA pH 8.2) to obtain a final volume of 10 µL. A volume of 2 µL of Blue 6X loading dye (Fisher Scientific) was added, after which 12 µL was run on a 0.7% wt/vol agarose gel (50 V) in the same TE buffer. DNA was visualized with SYBER Safe (Life Technologies).

## 4. Conclusions

Based on an acyl-hydrazone dynamic covalent chemistry that combined chiral arginine cationic units and fluorescent thiophene-ethynyl-fluorene π-conjugated units, two dynamic covalent polymers were formed in view of binding nucleic acids through ionic interactions. This self-assembly process by polycondensation results in the formation of DCP oligomers in DMSO depending on the concentration, as indicated by DOSY NMR spectroscopy and MALDI-TOF mass spectrometry.

When these DCPs are in presence of DNA in aqueous solutions, we observed the formation of supramolecular complexes whose chiroptical properties suggest folding/aggregation through π-stacking interactions. In addition, we have shown the possibility of forming fluorescent DCPs through DNA-templated self-assembly. This templating effect could possibly be helpful in view of monitoring the delivery functional nucleic acids, which will be the subject of further investigation.

Finally, distinct from more conventional approaches tagging a fluorescent/quencher group on DCPs [60], such an approach in which fluorescent groups are inserted within the main chain of DCPs precisely bears this unique opportunity of connecting the optical features with the nature of the self-assembly. Nevertheless, although this new example of aromatic polymers is able to bind nucleic acids and report on the complexation process by (chir)optical changes, much work remains to be conducted to achieve a (chiro)optical output which is specific and more sensitive to the nature of the templating nucleic acids. In this respect, further investigations will be devoted to the development of new fluorescent building blocks, displaying Aggregation-Induced Emission (AIE) properties, which may lead to more significant optical changes upon DNA binding.

## Figures and Tables

**Table 1:** Characterization of polycondensation reactions by DOSY-NMR. The hydrodynamic radius was determined using the Stokes–Einstein equation.

Entry	Compounds	Concentration (mM)	Diffusion Coefficient (m^2^ s^−1^)	Hydrodynamic Radius (Å)
1	**AFT^n^**	1 mM	7.104 × 10^−11^	15
2		10 mM	3.749 × 10^−11^	29
3		50 mM	2.886 × 10^−11^	37
4	**AFT^c^**	10 mM	4.445 × 10^−11^	24

**Table 2:** Characteristic values of optical properties of the fluorescent building blocks in DMSO.

Compound	λ_max_ (nm)	ε (L.mol^−1^.cm^−1^)	λ_em_ (nm)	ϕ_F_
**FT^n^**	398	37,600	481	0.54
**FT^c^**	394	38,100	487	0.50

## Data Availability

Data is available in the Supplementary Material.

## Supplementary Materials

The following supporting information can be downloaded at: https://www.mdpi.com/article/10.3390/molecules27196648/s1, Figure S1: FTIR-ATR spectrum of **FT**; Figure S2: ^1^H NMR spectrum (500 MHz) of **FT** in CDCl_3_ at 298K; Figure S3: ^13^C{^1^H} NMR spectrum (126 MHz) of **FT** in CDCl_3_ at 298K; Figure S4: High resolution MALDI-TOF mass spectrum of **FT** in the positive mode; Figure S5: FTIR-ATR spectrum of **FT^c^**; Figure S6: ^1^H NMR spectrum (500 MHz) of **FT^c^** in DMSO-*d*_6_ at 298K; Figure S7: ^13^C{^1^H} NMR spectrum (126 MHz) of **FT^c^** in DMSO-*d*_6_ at 298K; Figure S8: High resolution MALDI-TOF mass spectrum of **FT^c^** in the positive mode; Figure S9: FTIR-ATR spectrum of **FT^n^**; Figure S10: ^1^H NMR spectrum (500 MHz) of **FT^n^** in CDCl_3_ at 298K; Figure S11: ^13^C{^1^H} NMR spectrum (126 MHz) of **FT^n^** in CDCl_3_ at 298K; Figure S12: High resolution ASAP^+^ mass spectrum of **FT^n^** in the positive mode; Figure S13: Superimposition of DOSY-NMR spectra (600 MHz) of **AFT^n^** at 1 mM (green), 10 mM (purple) and 50 mM (pink) compared to **AFT^n^** (blue) in DMSO-*d*_6_ at 298K; Figure S14: Superimposition of DOSY-NMR spectra (600 MHz) of **AFT^c^** at 10 mM (blue) compared to **FT^c^** (green) in DMSO-*d*_6_ at 298K; Figure S15: MALDI-TOF (HCCA matrix) mass spectrometry analysis of **AFT^n^**, prepared by the self-assembly of **FT^n^** and **Ox-Arg-Hyd** carried out at 100 mM in DMSO. Figure S16: ^1^H NMR spectrum (600 MHz) of **AFT^n^** at 100 mM in DMSO-*d*_6_ at 298K; Figure S17: Synthesis of **FT^n^**-based model compounds from ***l*-Arg-Hyd** and ***d*-Arg-Hyd**; Figure S18: LCMS chromatogram of ***L*-1**; Figure S19: A/ UV-Visible absorption, B/ CD and C/ emission spectra (λ_exc_ = 385 nm) of **AFT^c^** at 10 μM in TE buffer after successive additions of guanidinium chloride; Figure S20: A/ UV-Visible absorption, B/ CD and C/ emission spectra (λ_exc_ = 385 nm) of **AFT^c^** at 10 μM in TE buffer after successive additions of ammonium sulfate; Figure S21: CD spectra of A/ **AFT^c^** and B/ **AFT^n^** solutions at 10 µM in TE buffer recorded from 20 °C to 86 °C; Table S1: Name, length and sequence of the dsDNAs employed in this study; Figure S22: UV-Vis absorption (A), CD (B) and emission (C) spectra of 10 µM **AFT^n^** solutions in TE buffer (pH 7.4) upon addition of calf thymus DNA; Figure S23: UV-Vis absorption (A), CD (B) and emission (C) spectra of 10 µM **AFT^c^** solutions in TE buffer (pH 7.4) upon addition of dsR43 DNA; UV-Vis absorption (D). CD (E) and emission (F) spectra of 10 µM **AFT^c^** solutions in TE buffer (pH 7.4) upon addition of calf thymus (*CT*) DNA; Figure S24: CD spectra of a mixture of **FT^n^** and **OxArgHyd** in TE buffer at 10 µM associated with calf thymus DNA at N/P = 5 recorded between 0 and 72 h A/ in absence and B/ presence of 100 equivalents of methoxyamine; Scheme S1: Formation of the oxime **Ox-FT^n^-Ox**.

## Sample Availability

Samples of the compounds might be available from the authors.
