# Supplementary material for: Fluorescent Dynamic Covalent Polymers for DNA Complexation and Templated Assembly
Source: Molecules. 2022 Oct 6;27(19):6648. doi: 10.3390/molecules27196648 (PMC9570939; doi:10.3390/molecules27196648)
Supplement: Supplementary file 1 [file molecules-27-06648-s001.zip › molecules-1861647-supplementary.pdf]

Fluorescent Dynamic Covalent Polymers for DNA Complexation and Templated Assembly

Clément Kotras,[1,2⊥] Maxime Leclercq,[1] Maxime Roger,[2] Camille Bouillon,[3] Antonio Recupido,[3] Aurélien Lebrun,[4] Yannick Bessin,[3] Philippe Gerbier,[2] Sébastien Richeter,[2] Sébastien Ulrich,[3]* Sébastien Clément[2]*, and Mathieu Surin[1]*

[1] Laboratory for Chemistry of Novel Materials, Center of Innovation and Research in Materials and Polymers (CIRMAP), University of Mons-UMONS, 7000 Mons, Belgium

[2] ICGM Institut Charles Gerhardt Montpellier, UMR 5253, CNRS, Université de Montpellier, ENSCM, 34095 Montpellier, France

[3] IBMM, Université de Montpellier, CNRS, ENSCM, Montpellier, France

[4] Laboratoire de Mesures Physiques, Montpellier, France

⊥ Present address: IES Institut d'Electronique et des Systèmes, UMR 5214, Université de Montpellier 34095 Montpellier, France

Table of contents

Figure S1: FTIR-ATR spectrum of **FT**. S3

Figure S2: ^{1}H NMR spectrum (500 MHz) of **FT** in $CDCl_3$ at 298K. S3

Figure S3: $^{13}C\{^{1}H\}$ NMR spectrum (126 MHz) of **FT** in $CDCl_3$ at 298K. S4

Figure S4: High resolution MALDI-TOF mass spectrum of **FT** in the positive mode S4

Figure S5: FTIR-ATR spectrum of **FT^{c}** S5

Figure S6: ^{1}H NMR spectrum (500 MHz) of **FT^{c}** in DMSO-d_6 at 298K. S5

Figure S7: $^{13}C\{^{1}H\}$ NMR spectrum (126 MHz) of **FT^{c}** in DMSO-d_6 at 298K S6

Figure S8: High resolution MALDI-TOF mass spectrum of **FT^{c}** in the positive mode S6

Figure S9: FTIR-ATR spectrum of **FT^{n}** S7

Figure S10: ^{1}H NMR spectrum (500 MHz) of **FT^{n}** in $CDCl_3$ at 298K S7

Figure S11: $^{13}C\{^{1}H\}$ NMR spectrum (126 MHz) of **FT^{n}** in $CDCl_3$ at 298K S8

Figure S12: High resolution ASAP^{+} mass spectrum of **FT^{n}** in the positive mode S8

Figure S13: Superimposition of DOSY-NMR spectra (600 MHz) of **AFT^{n}** at 1 mM (green), 10 mM (violet) and 50 mM (pink) compared to **AFT^{n}** (blue) in DMSO-d_6 at 298K S9

Figure S14: Superimposition of DOSY-NMR spectra (600 MHz) of **AFT**^{c} at 10 mM (blue) compared to **FT**^{c} (green) in DMSO-d_6 at 298K. S9

Figure S15: MALDI-TOF (HCCA matrix) mass spectrometry analysis of **AFT**^{n}, prepared by the self-assembly of **FT**^{n} and **Ox-Arg-Hyd** carried out at 100 mM in DMSO. S10

Figure S16: ^{1}H NMR spectrum (600 MHz) of **AFT**^{n} at 100 mM in DMSO-d_6 at 298K. S10

Figure S17: Synthesis of **FT**^{n}-based model compounds from ***L*-Arg-Hyd** and ***D*-Arg-Hyd**. S11

Figure S18: LCMS chromatogram of ***L*-1**. S11

Figure S19: A/ UV-Visible absorption, B/ CD and C/ emission spectra (λ_{exc} = 385 nm) of **AFT**^{c} at 10 μM in TE buffer after successive additions of guanidinium chloride. S12

Figure S20: A/ UV-Visible absorption, B/ CD and C/ emission spectra (λ_{exc} = 385 nm) of **AFT**^{c} at 10 μM in TE buffer after successive additions of ammonium sulfate. S13

Figure S21: CD spectra of A/ **AFT**^{c} and B/ **AFT**^{n} solutions at 10 µM in TE buffer recorded from 20°C to 86°C. S14

Table S1**:** Name, length and sequence of the dsDNAs employed in this study S14

Figure S22: UV-Vis absorption (A), CD (B) and emission (C) spectra of 10 µM **AFT**^{n} solutions in Tris-EDTA buffer (pH 7.4) upon addition of calf thymus DNA. S15

Figure S23: UV-Vis absorption (A), CD (B) and emission (C) spectra of 10 µM **AFT**^{n} solutions in Tris-EDTA buffer (pH 7.4) upon addition of dsR(43) (left) and calf thymus DNA (right). S16

Figure S24: CD spectra of a mixture of **FT**^{n} and **OxArgHyd** in TE buffer at 10 μM associated with calf thymus DNA at N/P = 5 recorded between 0 and 72h A/ in absence and B/ presence of 100 equivalents of methoxyamine. S17

Scheme S1: Formation of the oxime **Ox-FT**^{n}**-Ox**. S17

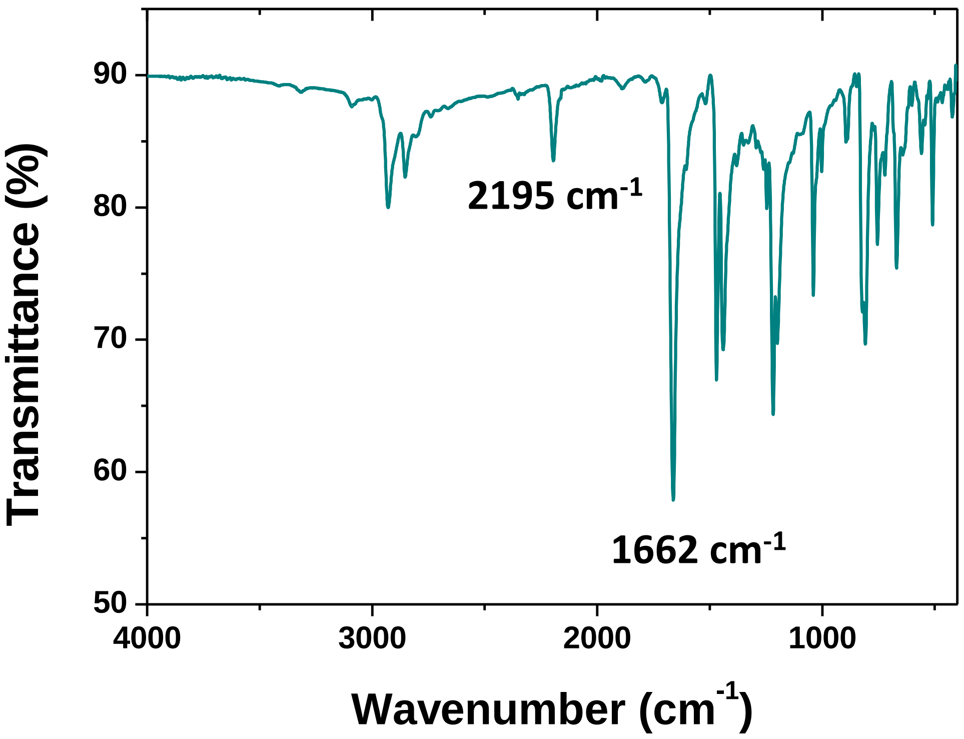

Figure S1: FTIR-ATR spectrum of **FT**.

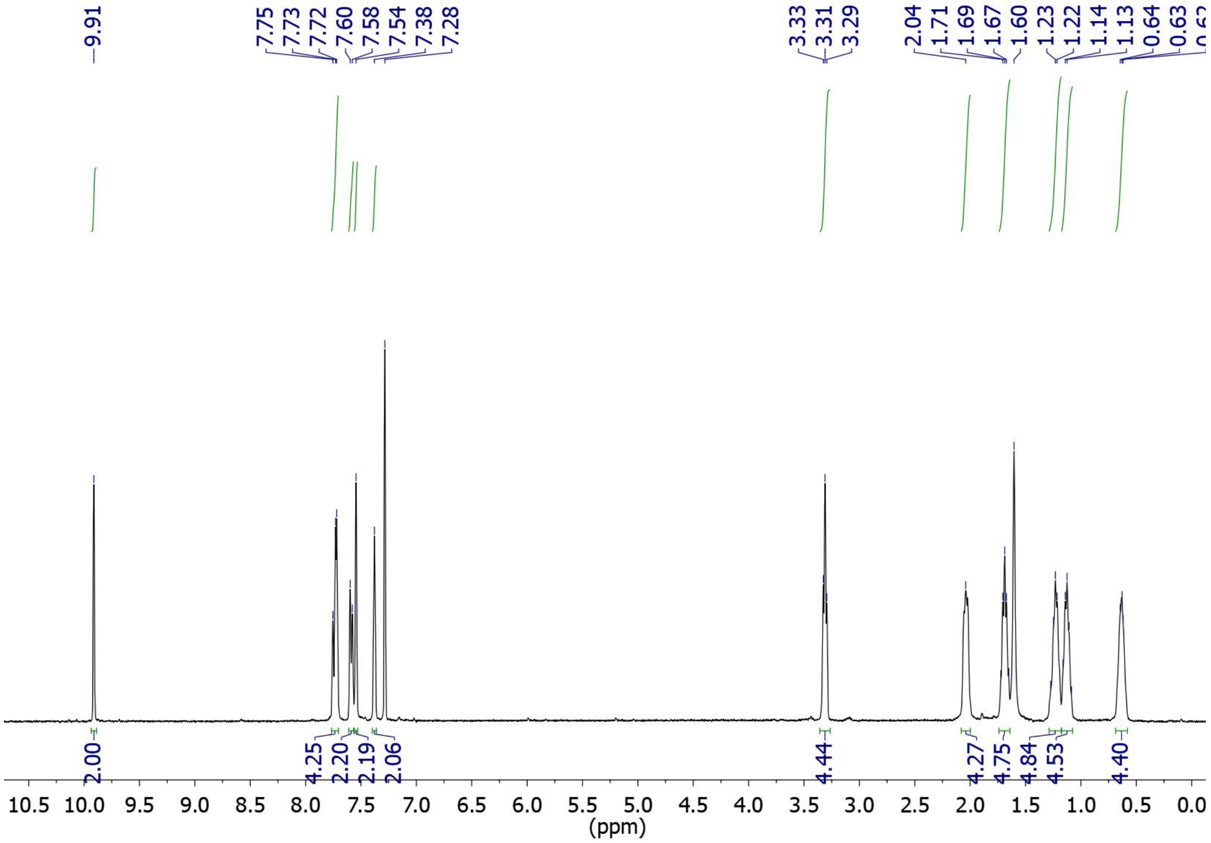

Figure S2: ^{1}H NMR spectrum (500 MHz) of **FT** in $CDCl_3$ at 298K.

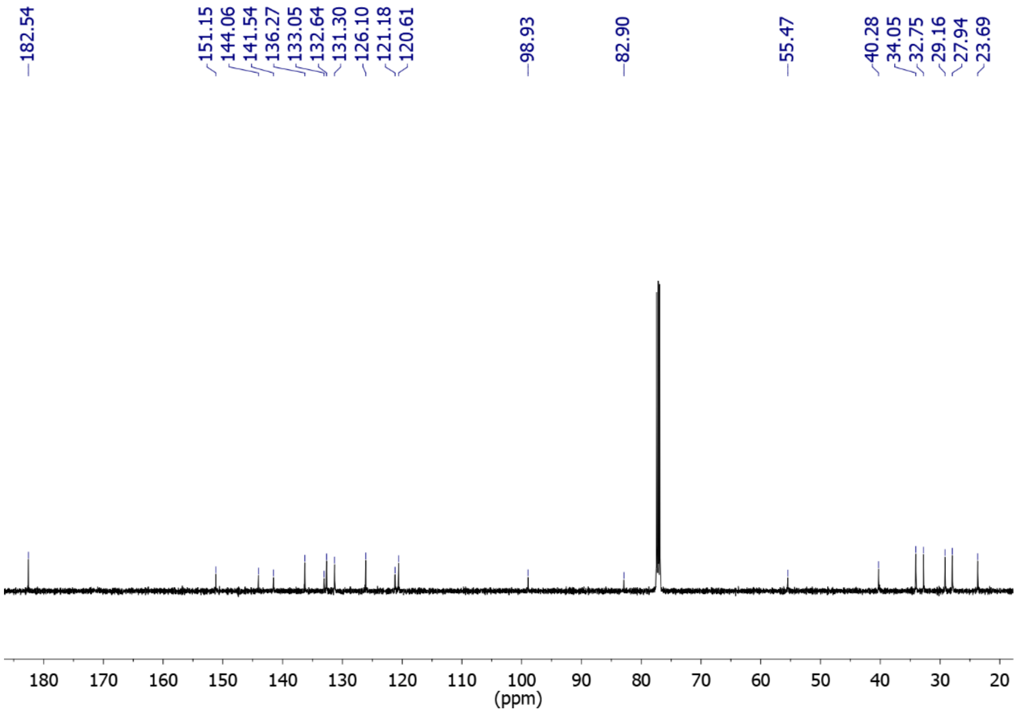

Figure S3: $^{13}C\{^{1}H\}$ NMR spectrum (126 MHz) of **FT** in $CDCl_3$ at 298K.

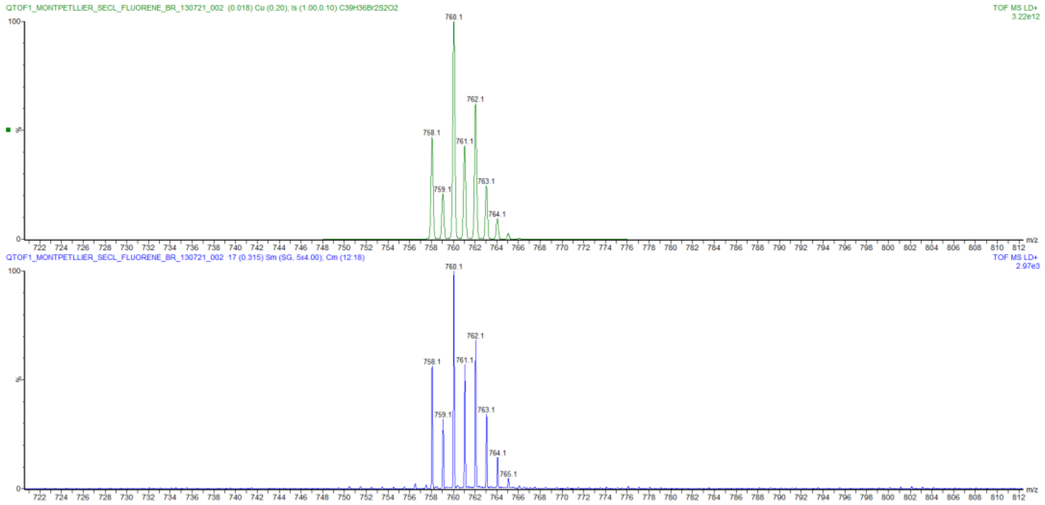

Elemental Composition Report

Single Mass Analysis
Tolerance = 25.0 PPM / DBE: min = -1.5, max = 150.0
Element prediction: Off
Number of isotope peaks used for i-FIT = 3

Monoisotopic Mass, Odd and Even Electron Ions
58 formula(e) evaluated with 4 results within limits (up to 50 closest results for each mass)
Elements Used:
C: 20-50 H: 0-60 O: 0-10 S: 2-2 Br: 2-2

Minimum:				-1.5				
Maximum:			5.0	25.0	150.0			
Mass	Calc. Mass	mDa	PPM	DBE	i-FIT	Norm	Conf(%)	Formula
758.0525	758.0523	0.2	0.3	21.0	78.0	0.254	77.53	C39 H36 O2 S2 Br2
	758.0582	-5.7	-7.5	12.0	79.8	1.995	13.61	C32 H40 O7 S2 Br2
	758.0430	9.5	12.5	8.0	81.3	3.500	3.02	C28 H40 O10 S2 Br2
	758.0371	15.4	20.3	17.0	80.6	2.840	5.84	C35 H36 O5 S2 Br2

Figure S4: High resolution MALDI-TOF mass spectrum of **FT** in the positive mode.

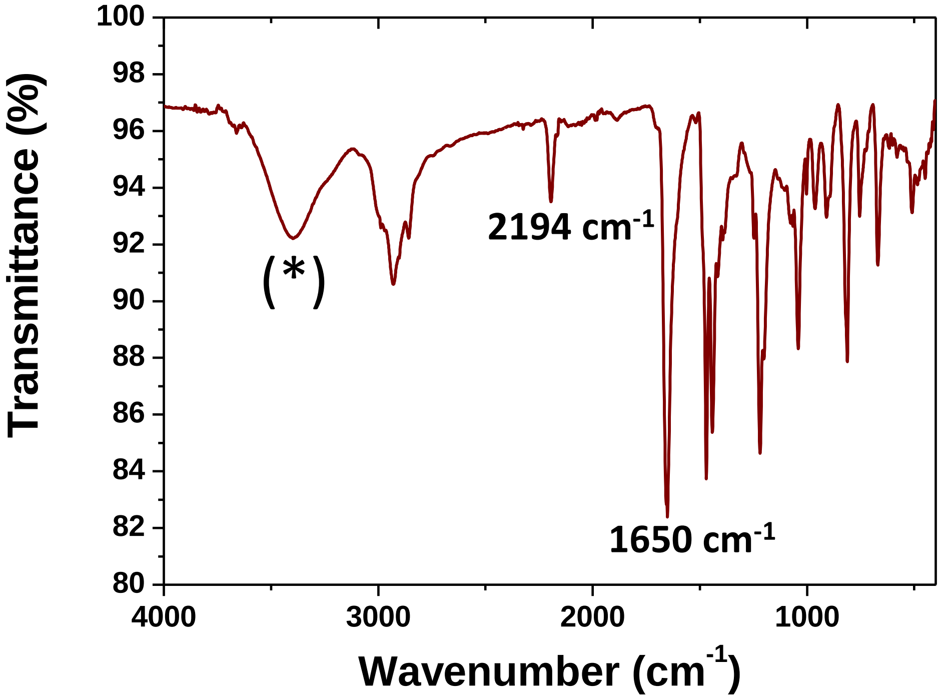

Figure S5: FTIR-ATR spectrum of **FT**[c]. (*) Traces of water due to the strong hydrophilicity of **FT**[c].

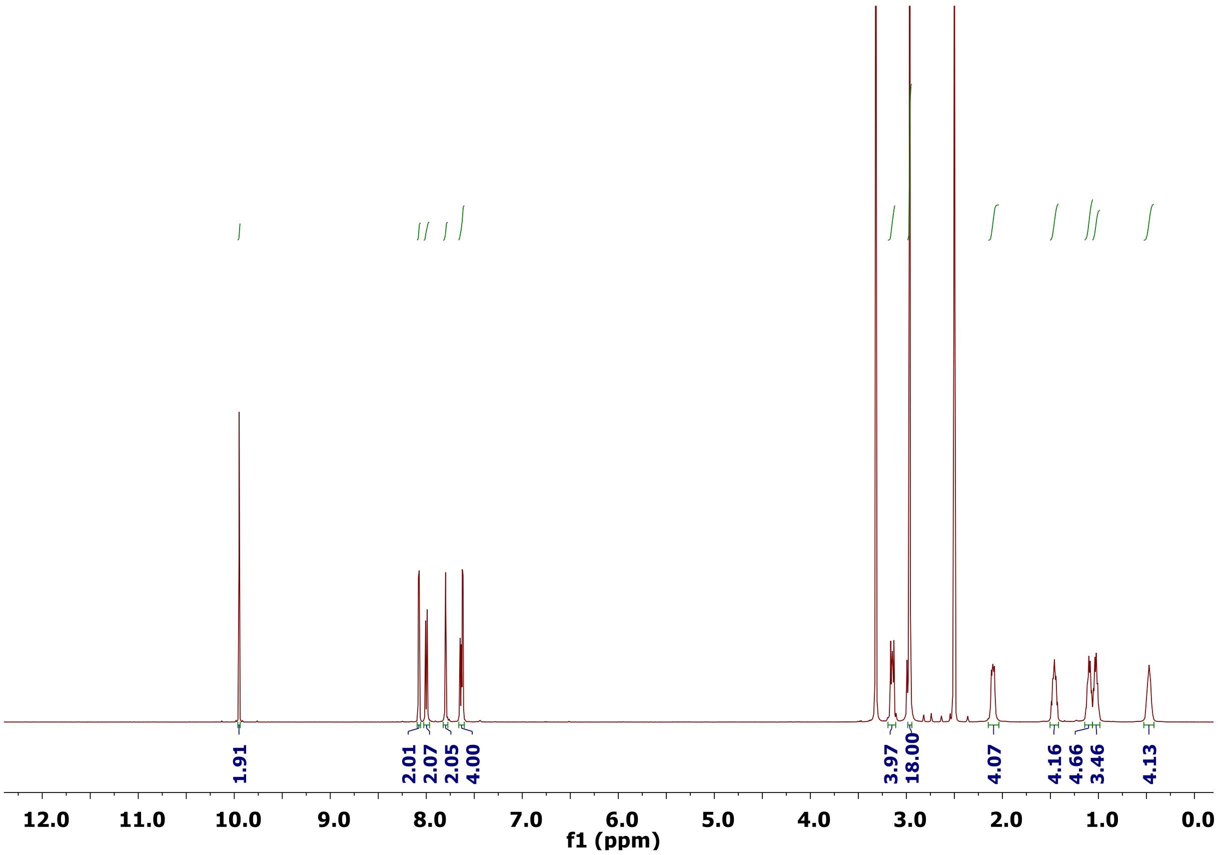

Figure S6: ^{1}H NMR spectrum (500 MHz) of **FT**[c] in DMSO-d_6 at 298K.

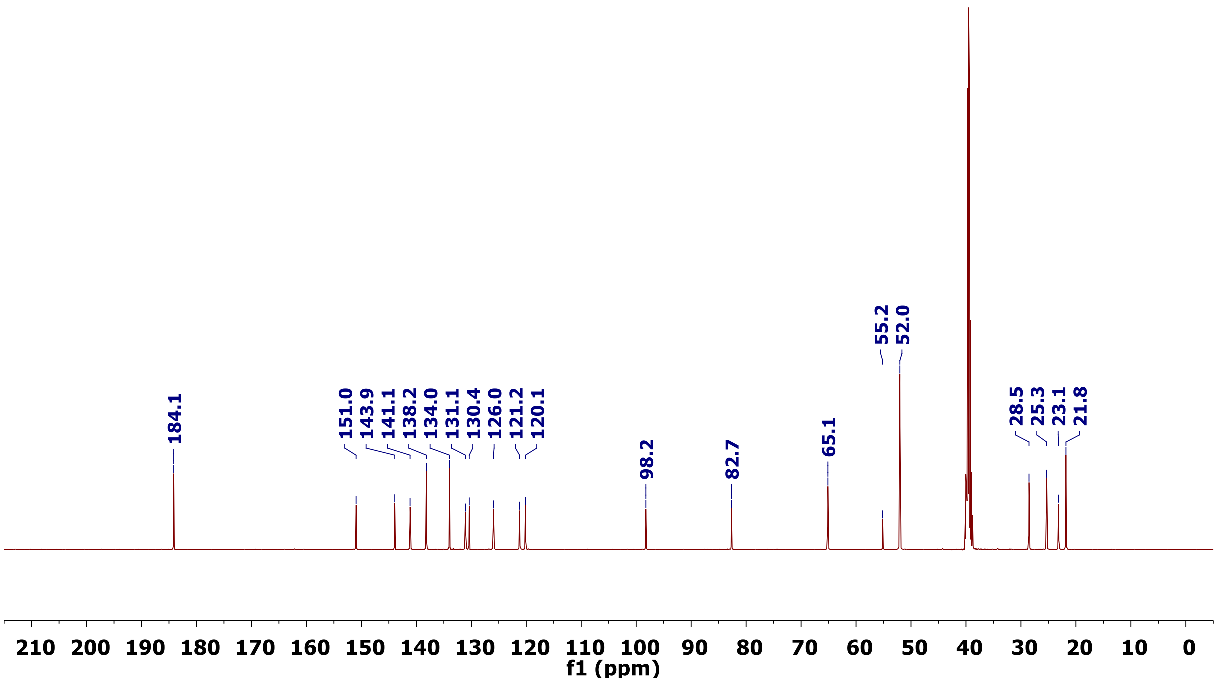

Figure S7: $^{13}C\{^{1}H\}$ NMR spectrum (126 MHz) of **FT**^{c} in DMSO-d_6 at 298K.

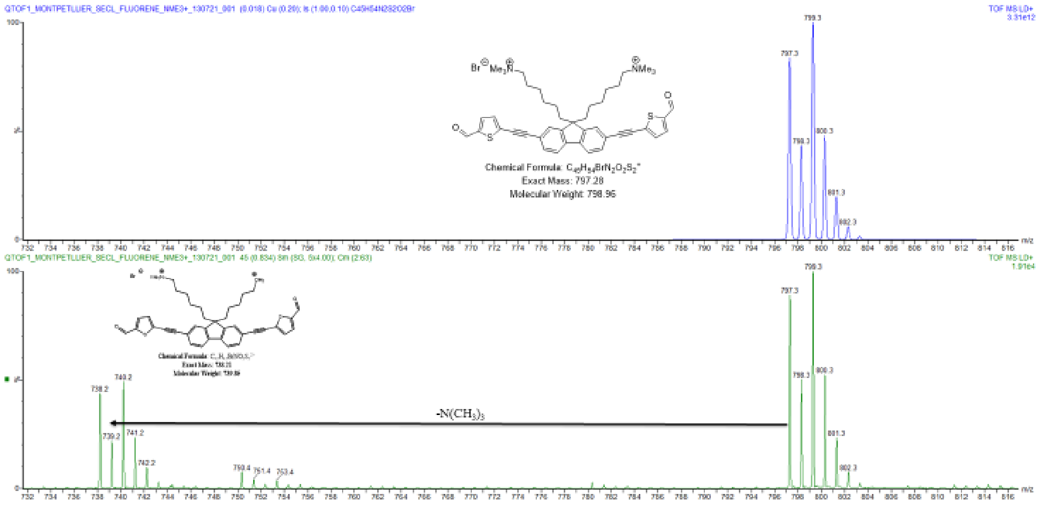

Elemental Composition Report

Single Mass Analysis
Tolerance = 25.0 PPM / DBE: min = -1.5, max = 150.0
Element prediction: Off
Number of isotope peaks used for i-FIT = 3

Monoisotopic Mass, Odd and Even Electron Ions
111 formula(e) evaluated with 4 results within limits (up to 50 closest results for each mass)
Elements Used:
C: 20-50 H: 0-60 N: 2-2 O: 0-10 S: 2-2 79Br: 1-2

Minimum:				-1.5				
Maximum:			5.0	25.0	150.0			
Mass	Calc. Mass	mDa	PPM	DBE	i-FIT	Norm	Conf(%)	Formula
797.2805	797.2810	-0.5	-0.6	19.5	107.1	1.553	21.15	C45 H54 N2 O2 S2 79Br
	797.2869	-6.4	-8.0	10.5	106.7	1.214	29.71	C38 H58 N2 O7 S2 79Br
	797.2716	8.9	11.2	6.5	106.6	1.095	33.46	C34 H58 N2 O10 S2 79Br
	797.2658	14.7	18.4	15.5	107.4	1.852	15.68	C41 H54 N2 O5 S2 79Br

Figure S8: High resolution MALDI-TOF mass spectrum of **FT**^{c} in the positive mode.

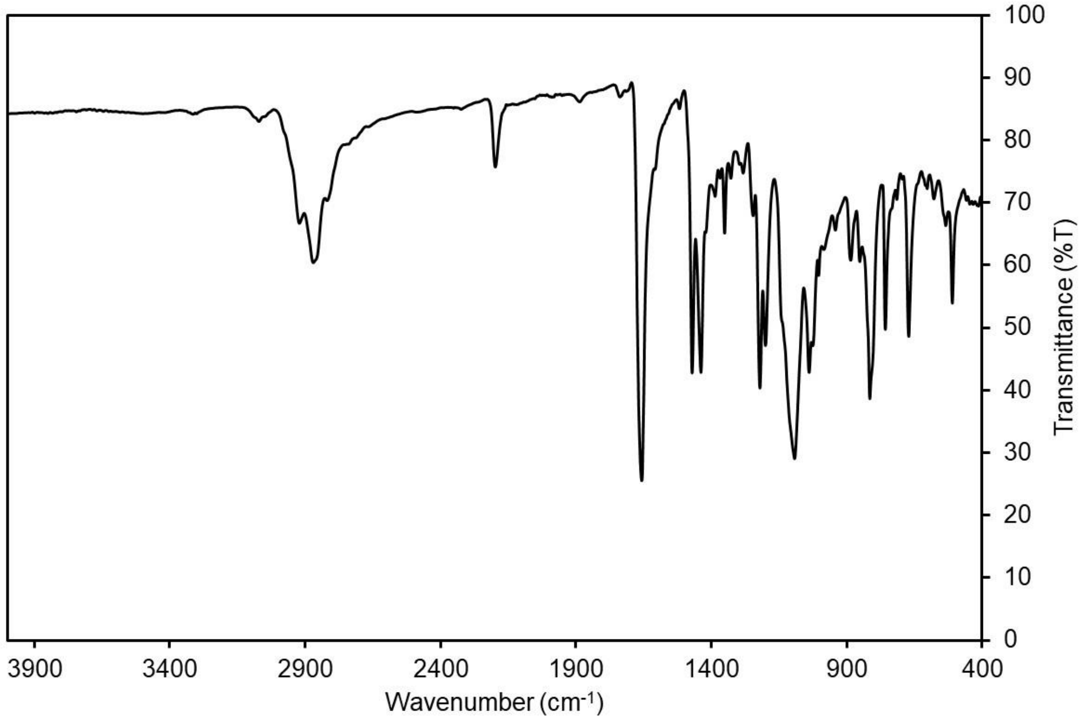

Figure S9: FTIR-ATR spectrum of **FT**^{n}.

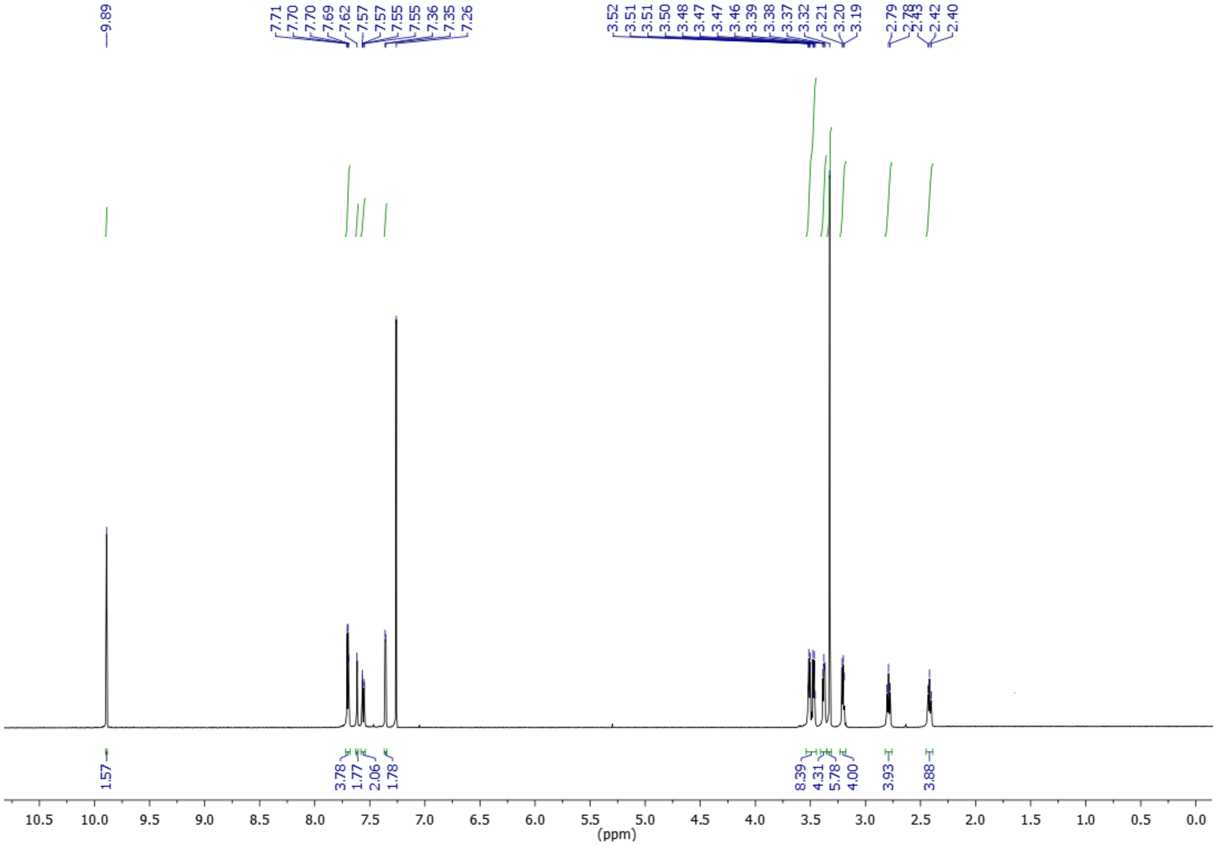

Figure S10: ^{1}H NMR spectrum (500 MHz) of **FT**^{n} in $CDCl_3$ at 298K.

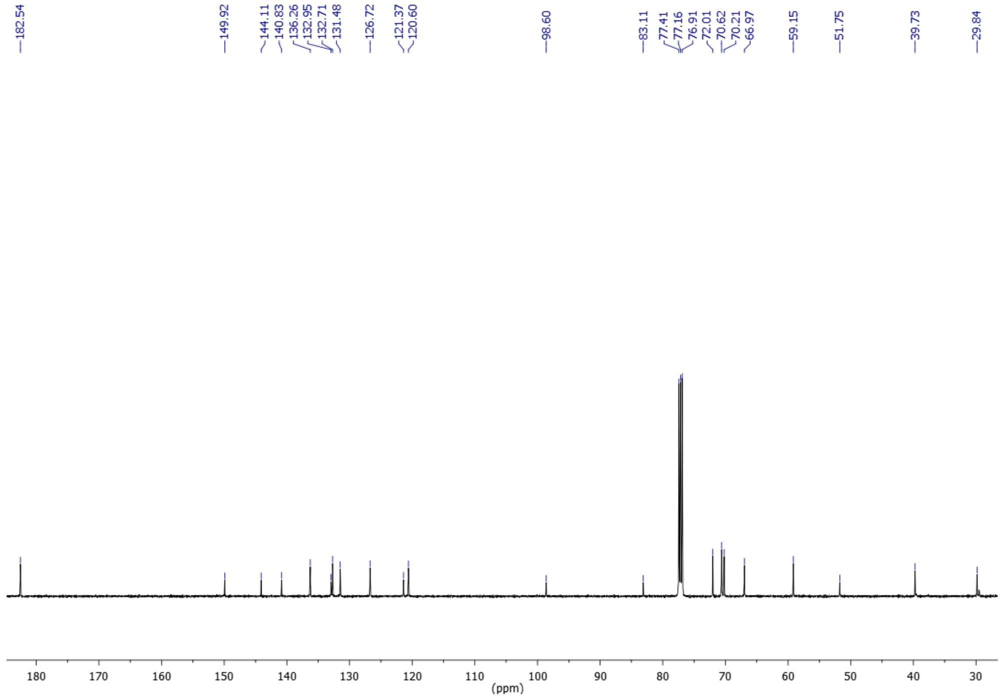

Figure S11: $^{13}C\{^{1}H\}$ NMR spectrum (126 MHz) of **FT^{n}** in $CDCl_3$ at 298K.

Single Mass Analysis

Tolerance = 3.0 PPM / DBE: min = -1.5, max = 100.0

Element prediction: Off

Number of isotope peaks used for i-FIT = 3

Monoisotopic Mass, Odd and Even Electron Ions

452 formula(e) evaluated with 2 results within limits (up to 50 best isotopic matches for each mass)

Elements Used:

C: 0-100 H: 0-100 O: 0-20 S: 0-2

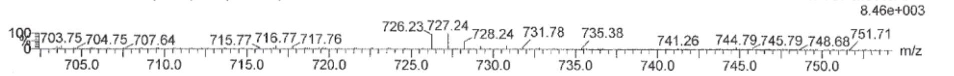

Minimum:				-1.5				
Maximum:		10.0	3.0	100.0				
Mass	Calc. Mass	mDa	PPM	DBE	i-FIT	Norm	Conf(%)	Formula
726.2314	726.2312	0.2	0.3	22.0	263.6	0.094	91.03	C40 H38 O13
	726.2321	-0.7	-1.0	21.0	265.9	2.411	8.97	C41 H42 O8 S2

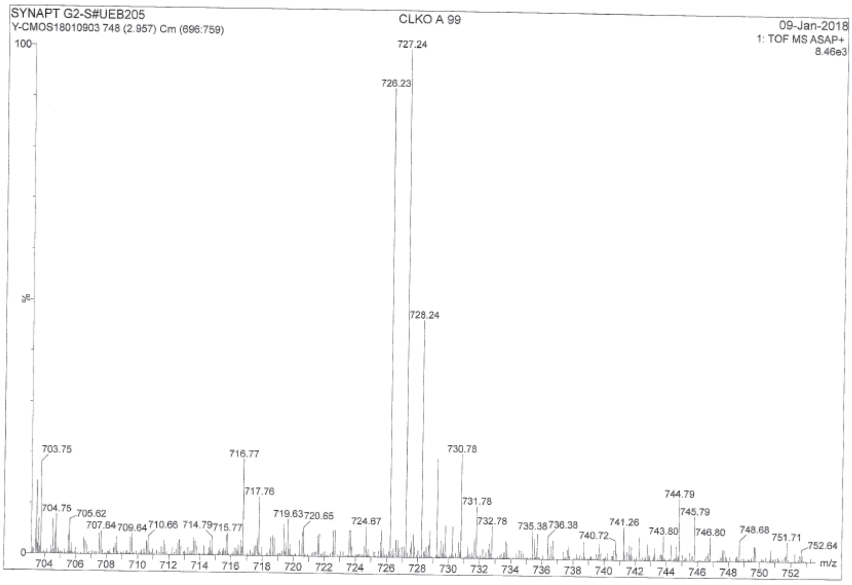

Figure S12: High resolution ASAP^{+} mass spectrum of **FT^{n}** in the positive mode**.**

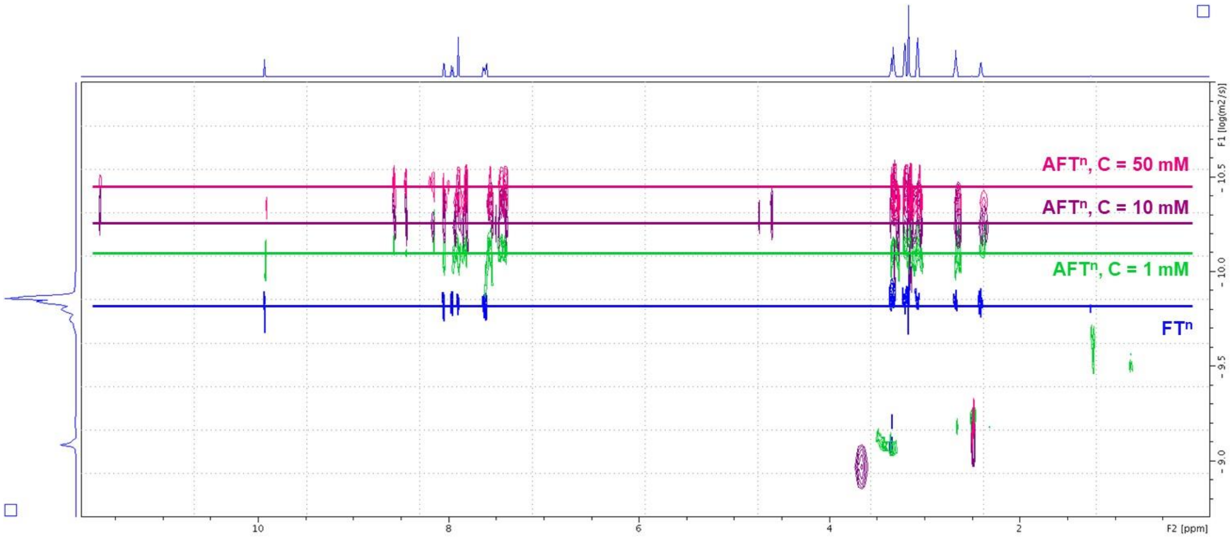

Figure S13: Superimposition of DOSY-NMR spectra (600 MHz) of **AFT^n** at 1 mM (green), 10 mM (purple) and 50 mM (pink) compared to **AFT^n** (blue) in DMSO-d_6 at 298K.

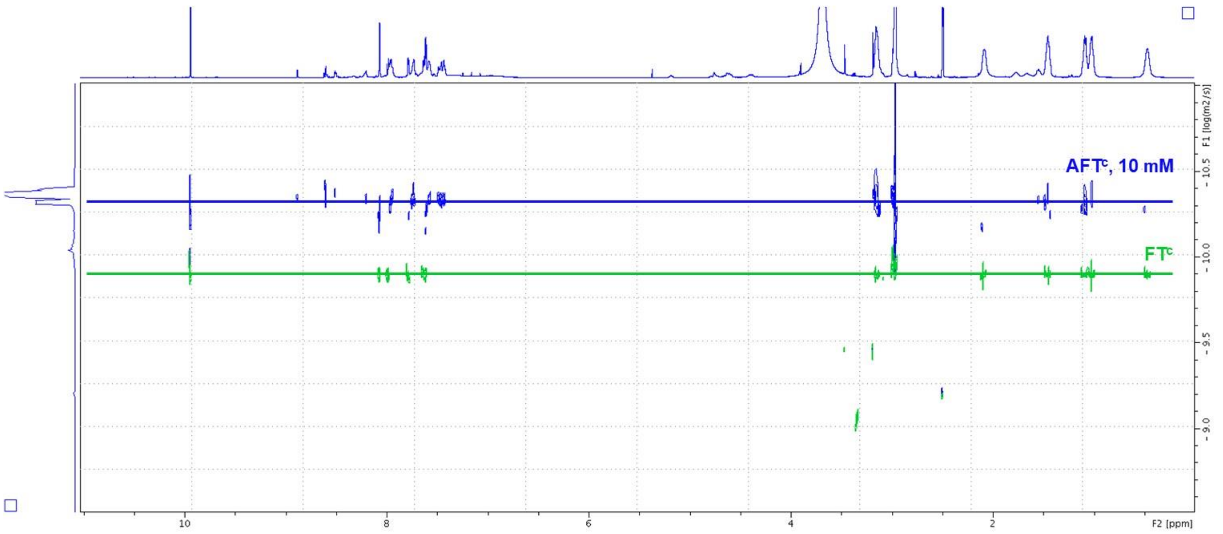

Figure S14: Superimposition of DOSY-NMR spectra (600 MHz) of **AFT^c** at 10 mM (blue) compared to **FT^c** (green) in DMSO-d_6 at 298K.

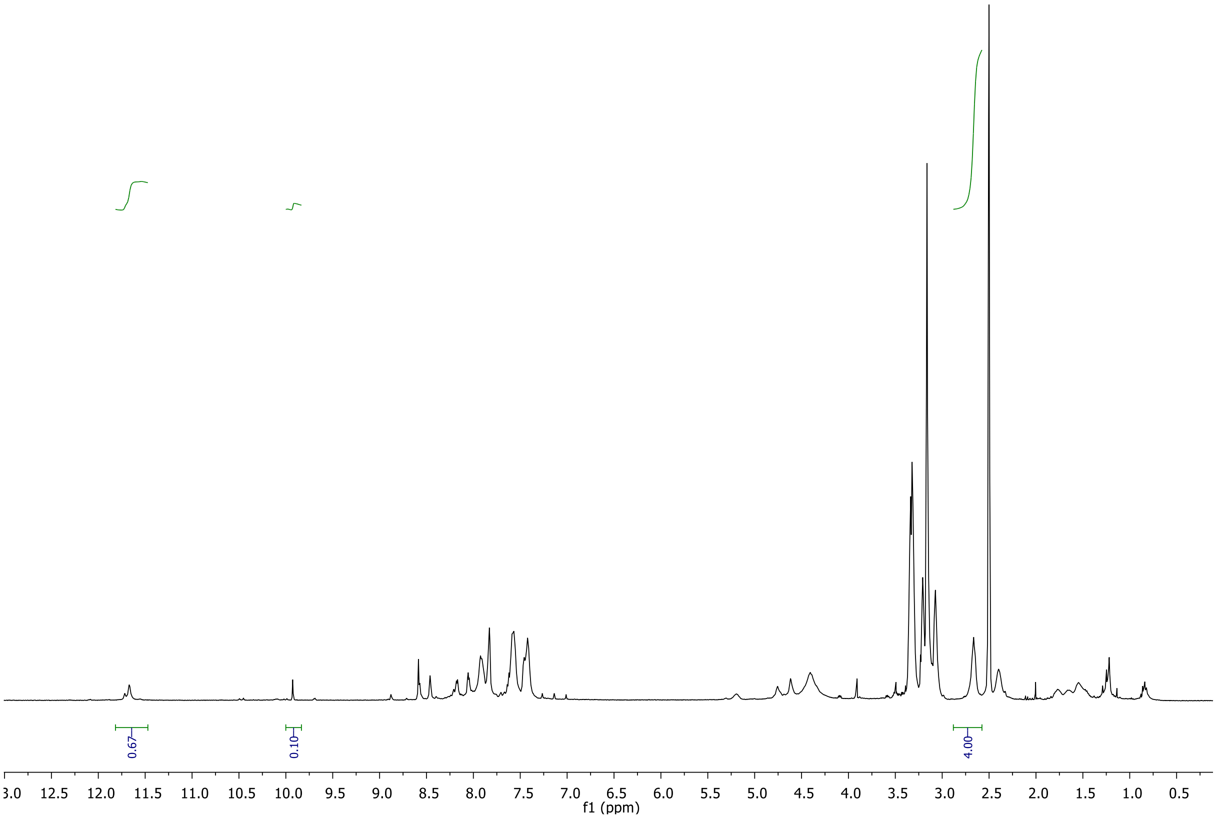

Figure S15: ^{1}H NMR spectrum (600 MHz) of **AFT^{n}** at 100 mM in DMSO-d_6 at 298K.

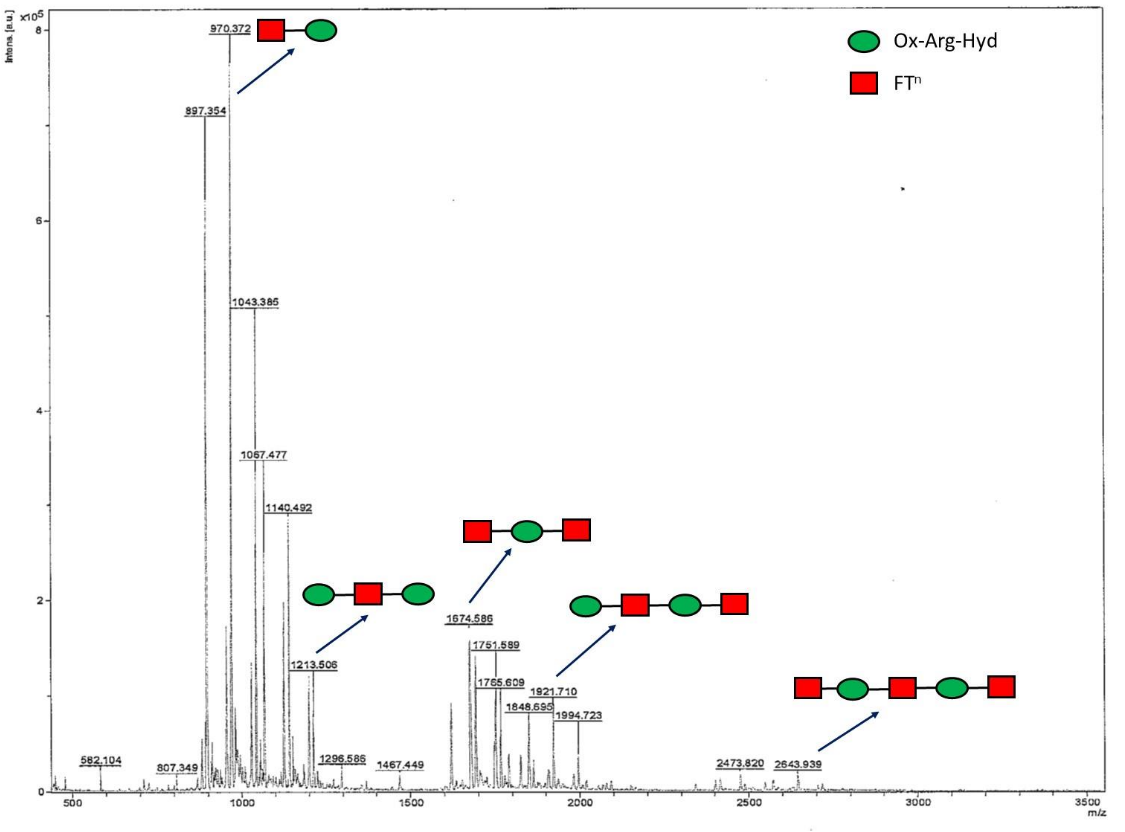

Figure S16: MALDI-TOF (HCCA matrix) mass spectrometry analysis of **AFT^{n}**, prepared by the self-assembly of **FT^{n}** and **Ox-Arg-Hyd** carried out at 100 mM in DMSO.

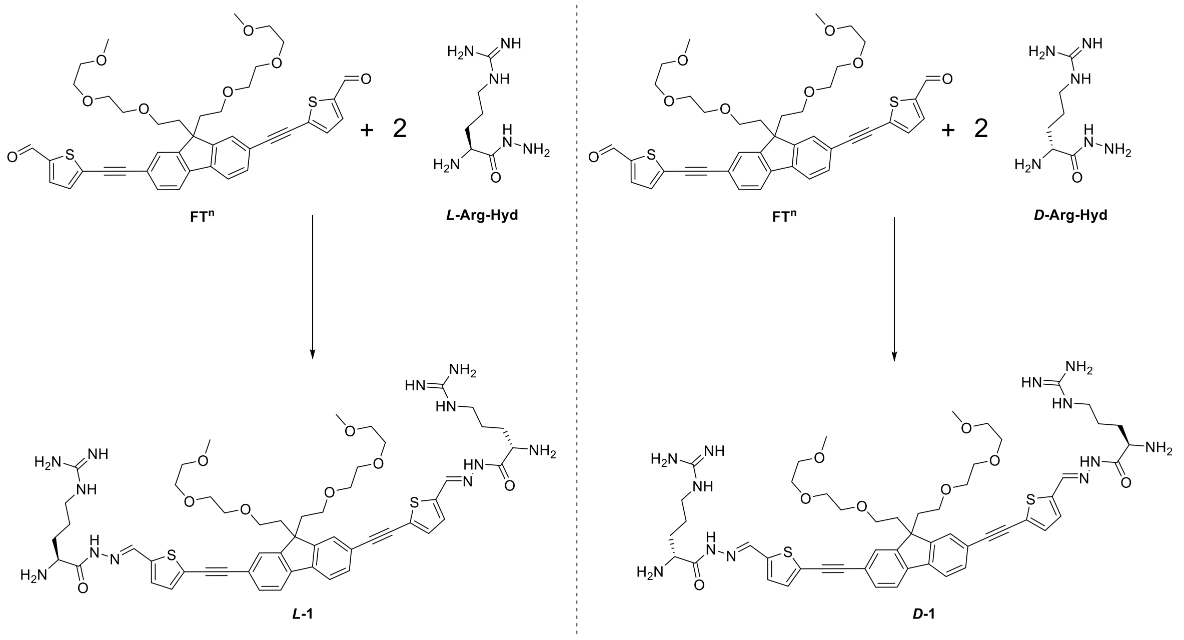

Figure S17: Synthesis of **FT^n**-based model compounds from ***L*-Arg-Hyd** and ***D*-Arg-Hyd**.

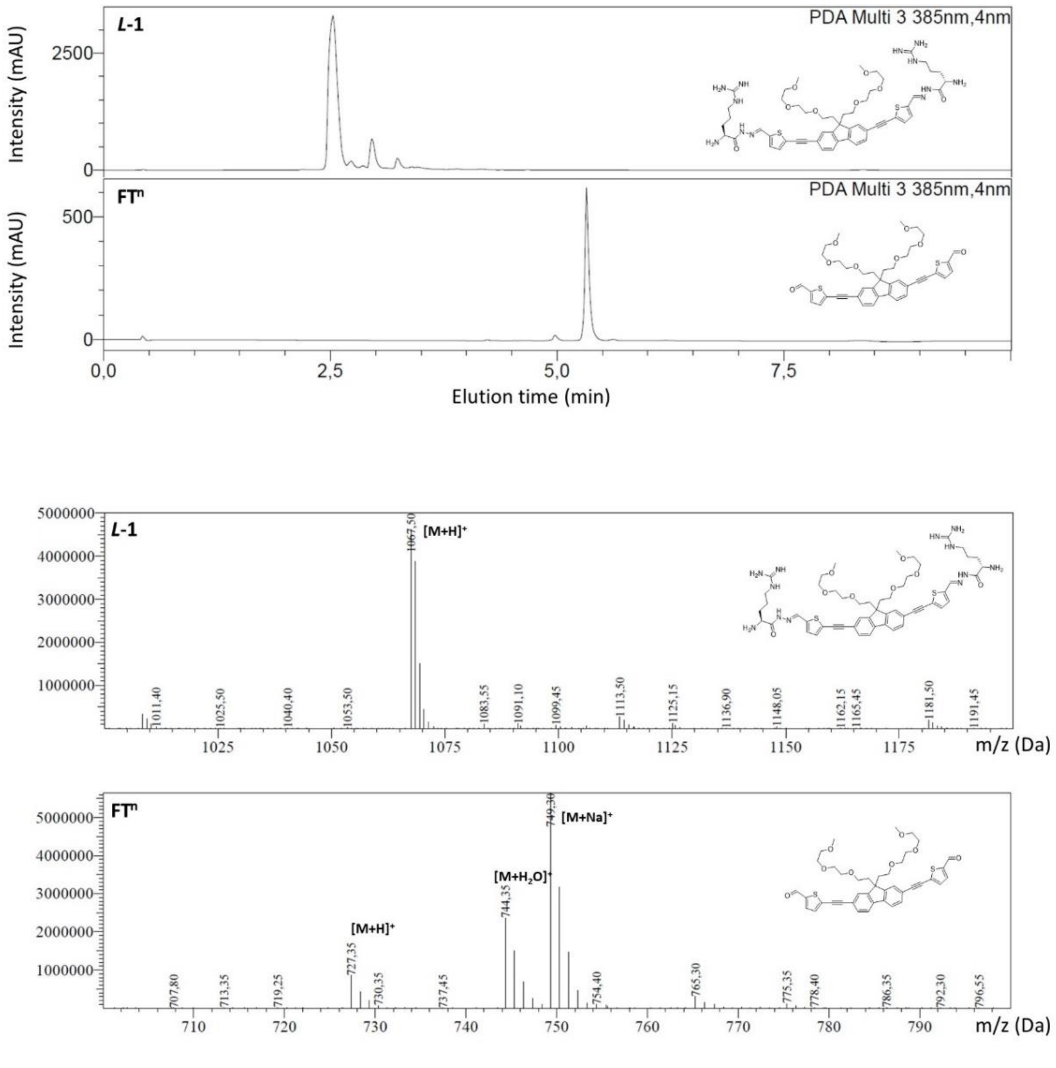

Figure S18: LCMS chromatogram of ***L*-1.**

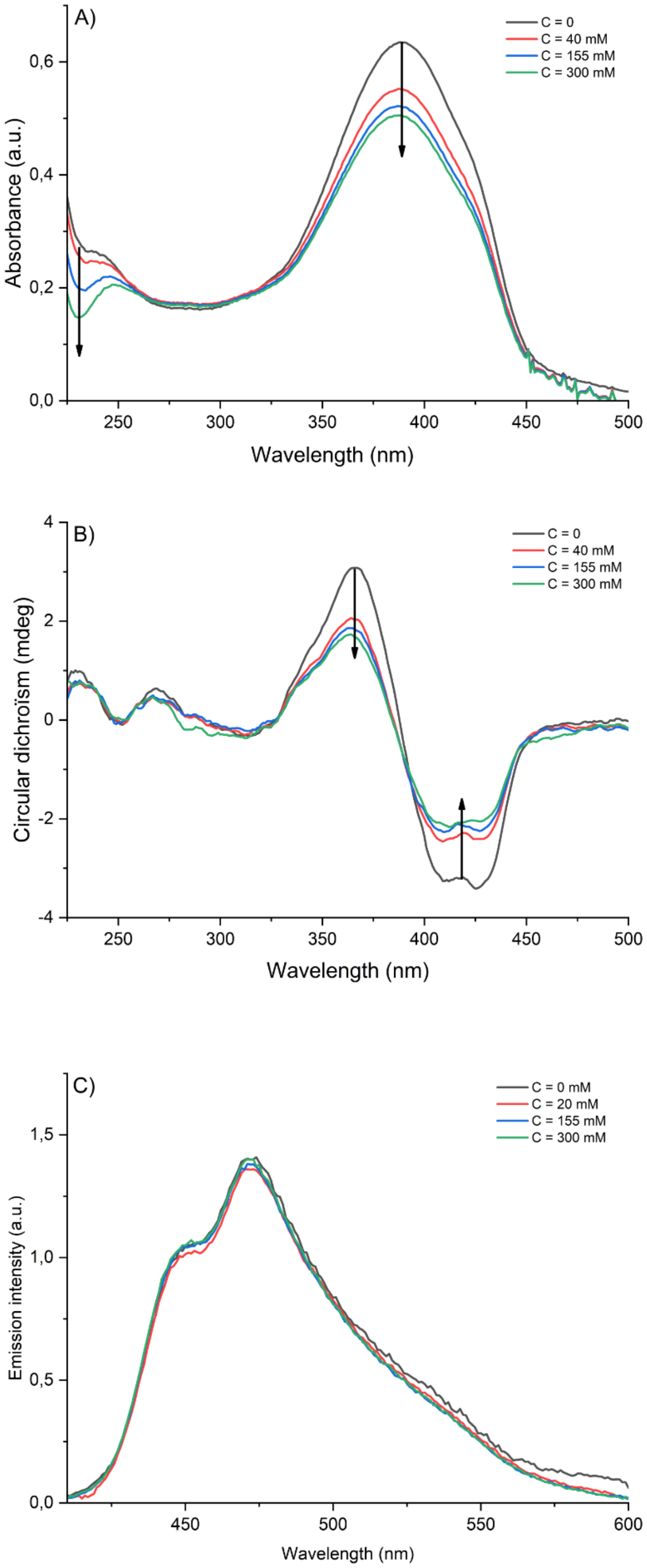

Figure S19: A) UV-Visible absorption, B) CD and C) emission spectra (λ_{exc} = 385 nm) of **AFT**^{c} at 10 μM in TE buffer after successive additions of guanidinium chloride.

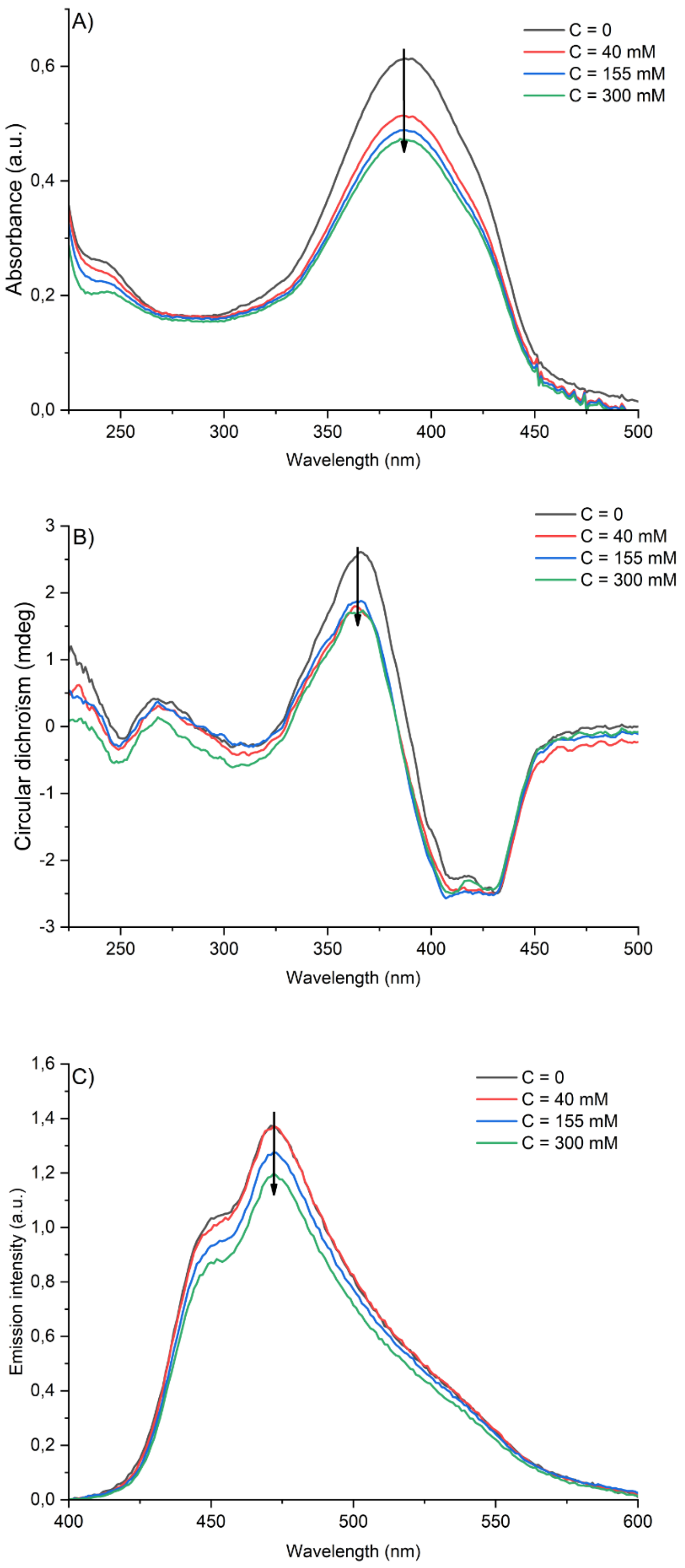

Figure S20: A) UV-Visible absorption, B) CD and C) emission spectra (λ_{exc} = 385 nm) of **AFT**^{c} at 10 μM in TE buffer after successive additions of ammonium sulfate.

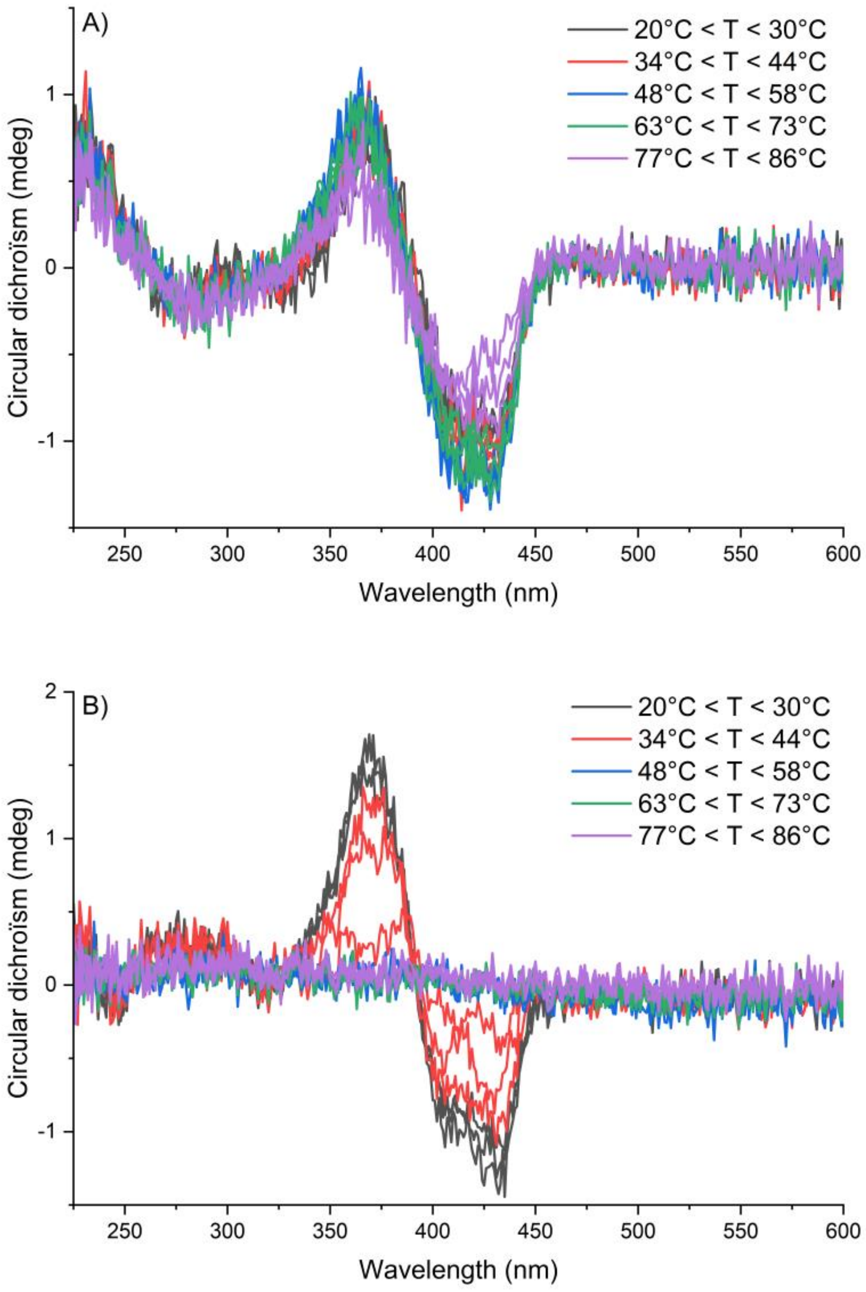

Figure S21: CD spectra of A) $\mathbf{AFT^c}$ and B) $\mathbf{AFT^n}$ solutions at 10 μM in TE buffer recorded from 20°C to 86°C.

Name	Length	Sequence (5'-3')
**dsR20**	20	5'-CGT CAC GTA AAT CGG TTA AC-3'
**dsR43**	43	5'-CGT CAC GTA AAT CGG TTA ACA AAT GGC TTT CGA AGC TAG CTT C-3'

Table S1**:** Name, length and sequence of the dsDNAs employed in this study.

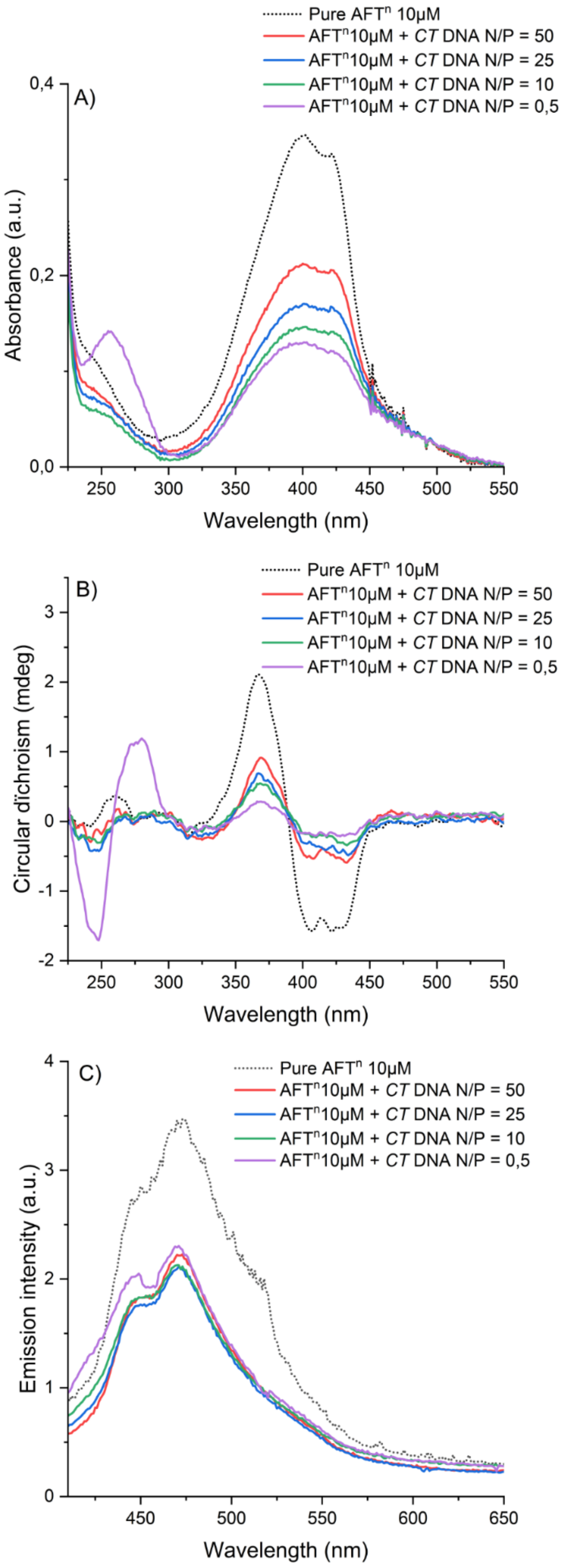

Figure S22: UV-Vis absorption (A), CD (B) and emission (C) spectra of 10 μM **AFT^{n}** solutions in TE buffer (pH 7.4) upon addition of calf thymus (*CT*) DNA.

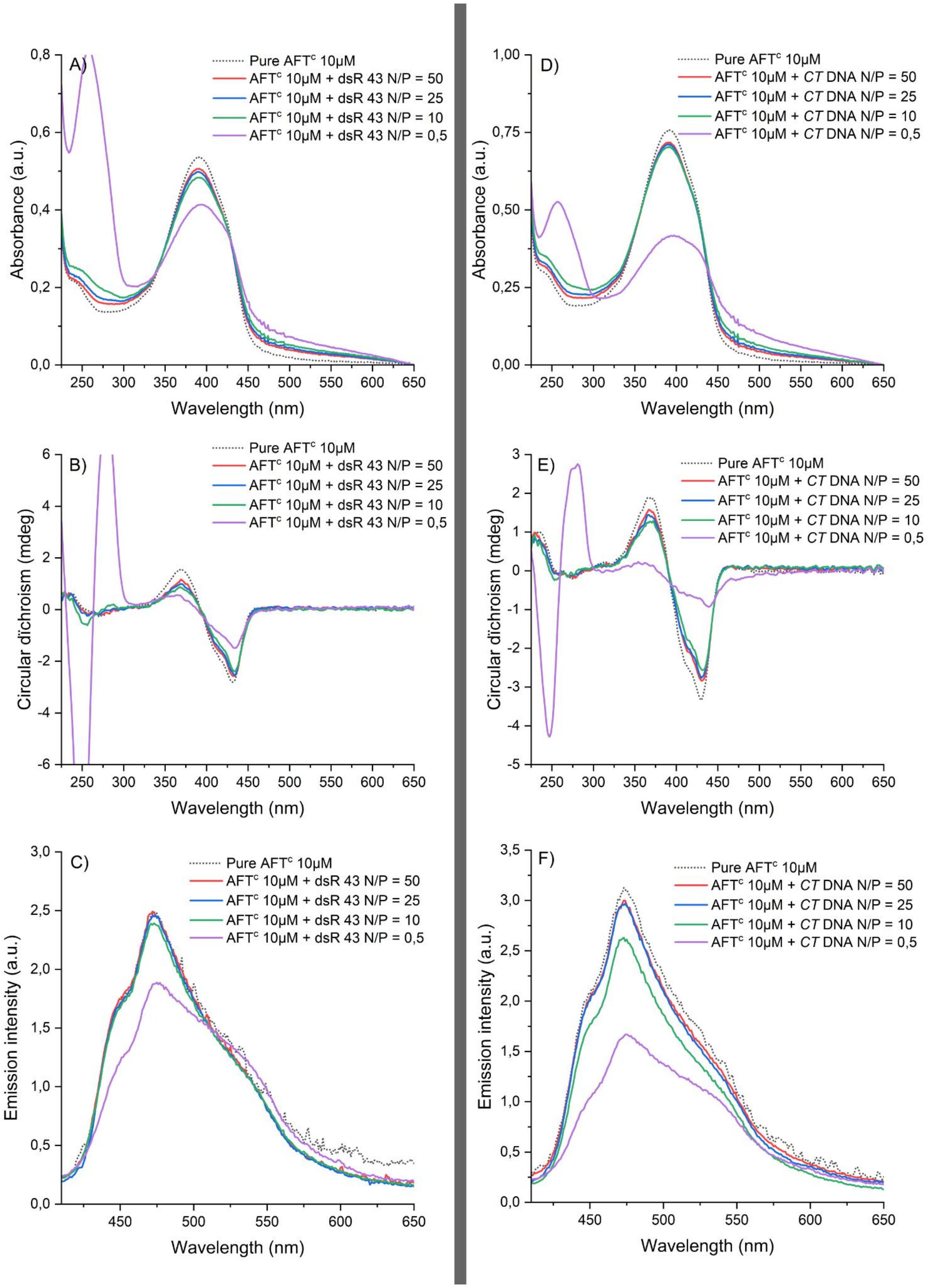

Figure S23: UV-Vis absorption (A), CD (B) and emission (C) spectra of 10 µM **AFT^c** solutions in TE buffer (pH 7.4) upon addition of dsR43 DNA ; UV-Vis absorption (D). CD (E) and emission (F) spectra of 10 µM **AFT^c** solutions in TE buffer (pH 7.4) upon addition of calf thymus (*CT*) DNA.

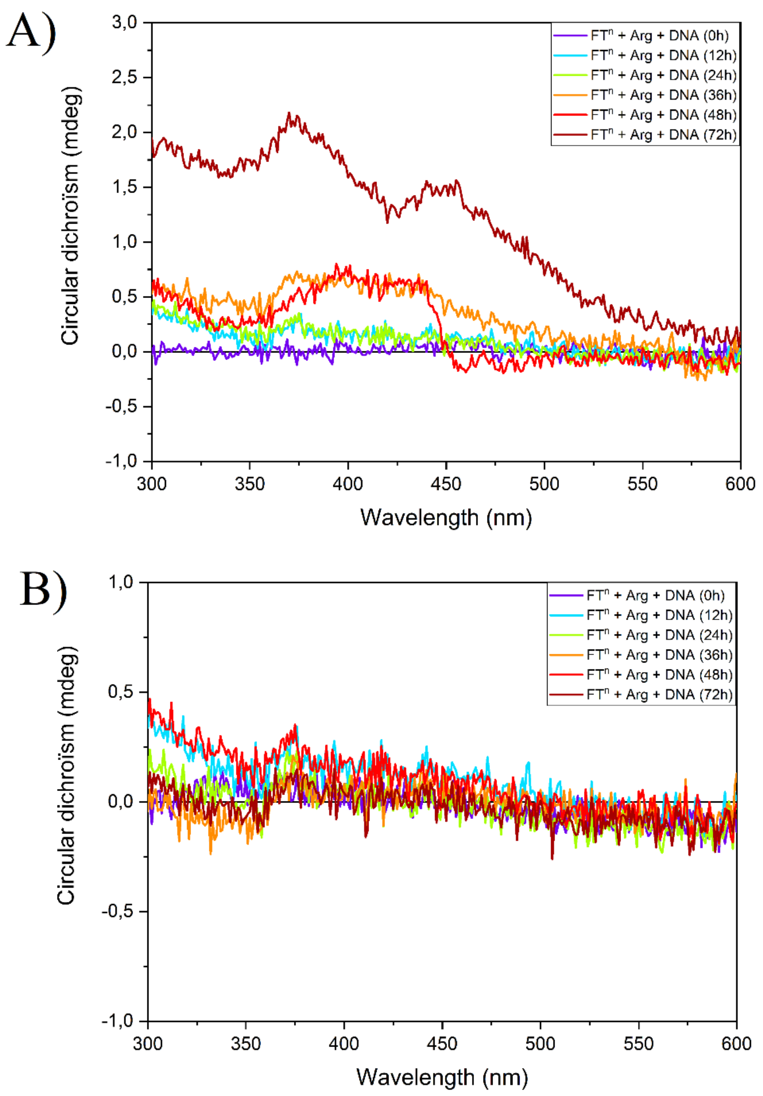

Figure S24**:** CD spectra of a mixture of **FT**^{n} and **OxArgHyd** in TE buffer at 10 µM associated with calf thymus DNA at N/P = 5 recorded between 0 and 72h A/ in absence and B/ presence of 100 equivalents of methoxyamine.

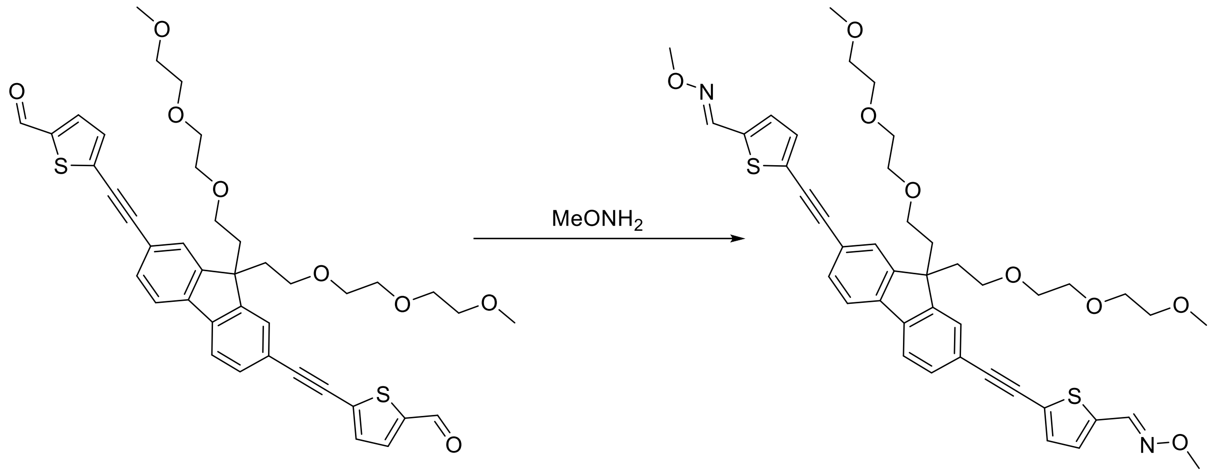

Scheme S1: Formation of the oxime **Ox-FT**^{n}**-Ox**.
